# Classification Under Local Differential Privacy with Model Reversal and Model Averaging

**Published:** 2026

**Authors:** Caihong Qin, Yang Bai

**Affiliations:** Department of Epidemiology and Biostatistics, School of Public Health, Indiana University, Indiana, USA; School of Statistics and Data Science & Institute of Data Science and Statistics, Shanghai University of Finance and Economics, Shanghai, China

**Keywords:** dataset utility, excess risk, functional data, private learning, transfer learning

## Abstract

Local differential privacy (LDP) has become a central topic in data privacy research, offering strong privacy guarantees by perturbing user data at the source and removing the need for a trusted curator. However, the noise introduced by LDP often significantly reduces data utility. To address this issue, we reinterpret private learning under LDP as a transfer learning problem, where the noisy data serve as the source domain and the unobserved clean data as the target. We propose novel techniques specifically designed for LDP to improve classification performance without compromising privacy: (1) a noised binary feedback-based evaluation mechanism for estimating dataset utility; (2) model reversal, which salvages underperforming classifiers by inverting their decision boundaries; and (3) model averaging, which assigns weights to multiple reversed classifiers based on their estimated utility. We provide theoretical excess risk bounds under LDP and demonstrate how our methods reduce this risk. Empirical results on both simulated and real-world datasets show substantial improvements in classification accuracy.

## 1. Introduction

Data privacy has become a paramount concern as technological innovations continue to permeate daily life. Differential Privacy (DP; Dwork et al., 2006) has emerged as a leading framework for privacy-preserving statistical analyses, providing a rigorous definition that limits the information an adversary can learn from released summaries. However, classical DP often assumes a trusted data curator who receives the raw data and applies privacy mechanisms. This assumption often fails in scenarios involving untrusted data collectors or highly sensitive information (Liu et al., 2022). Alternative privacy-preserving approaches such as cryptographic methods (Salami et al., 2023) and Homomorphic Encryption (Marcolla et al., 2022) have been considered. While these methods can protect raw data through encryption and secure computation, they typically introduce high computational overhead and do not inherently address all privacy risks from published statistics (Gong et al., 2024; Peng, 2019). By contrast, Local Differential Privacy (LDP; Kasiviswanathan et al., 2011) addresses the problem at the data source. Under LDP, each individual perturbs their own data before sending any information to the collector, thereby removing the need for a trusted curator. This decentralized approach can significantly mitigate privacy leakage and is relatively scalable in real-world systems. Currently, multiple variants of LDP exist (Geumlek and Chaudhuri, 2019; Erlingsson et al., 2020; Meehan et al., 2022; Huang and Canonne, 2022; Qin et al., 2023), with pure ε-LDP being the most widely used and well-established. Its formal definition is given as follows:

**Definition 1 (ε-LDP,** Kasiviswanathan et al. 2011**)** *A randomized mechanism* ℳ *is said to satisfy* ε*-local differential privacy (LDP) if, for any pair of input values* $u_{1},u_{2}$ *in the domain of* ℳ *and any possible output* v*, it holds that*

$$P\left(ℳ\left(u_{1}\right)=v\right)\leq e^{\varepsilon }\;P\left(ℳ\left(u_{2}\right)=v\right).$$

LDP has already been deployed by major technology companies such as Apple (Differential Privacy Team, 2017), Google (Erlingsson et al., 2014), and Microsoft (Ding et al., 2017). While it offers stronger privacy protects against untrusted data collectors, LDP often adds more noise per user than standard DP, potentially harming data utility. Balancing the privacy-utility trade-off is a key challenge, particularly in private learning scenarios, which involve training machine learning models under LDP constraints (Yang et al., 2020). This contrasts with much of the existing LDP literature, which focuses on simpler statistical queries (e.g., frequency counts or sums) (Wang et al., 2017).

Private learning under LDP faces two major difficulties (Ye and Hu, 2020). First, preserving correlations among features (Wang et al., 2019b) and between features and labels (Yilmaz et al., 2020) is critical for accurate model training. The noise required by LDP can disrupt these relationships, reducing model accuracy. Second, when data are high-dimensional, one may split the privacy budget ε across dimensions or randomly select a single dimension to report (Nguyên et al., 2016; Arcolezi et al., 2021, 2022). As the dimensionality d grows, the effective privacy budget per dimension drops drastically, deteriorating utility even further. Meanwhile, omitting certain dimensions altogether may cause incomplete information for learning.

These challenges motivate our work. We focus on the classification problem, aiming to fully exploit data utility and enhance classifier performance while maintaining the same level of LDP protection. By better leveraging noisy data, we aim to boost predictive accuracy on the true (unperturbed) distribution without sacrificing privacy. Equivalently, for a desired performance target, stronger privacy guarantees can be offered to data owners. We draw inspiration from transfer learning (Weiss et al., 2016; Hosna et al., 2022), which typically uses source data to aid learning on a target domain. Here, the noised (LDP-perturbed) data play the role of source data, and the unobserved true data serve as the target. However, two key distinctions arise:

- Unlike standard transfer learning, we lack direct observations from the target distribution, as only noised data are available under LDP.
- Negative transfer scenarios (Li et al., 2022) (where some source datasets could degrade performance) may be more frequent under LDP, because the noise injection can substantially distort the correlation information.

Although existing transfer learning techniques cannot be applied directly, we adapt several ideas to measure and exploit the transferability of noised datasets. Doing so enables us to assess dataset utility and design robust private-learning techniques. Below, we outline how our methods address the classic transfer learning questions (Pan and Yang, 2009)—what to transfer, when to transfer, and how to transfer—under LDP settings:

1. What to transfer. Our goal is to learn the relationship between the response and covariates. One standard approach under LDP collects noised feature-label pairs from clients for model training. In addition to this, we introduce a novel LDP mechanism in which a subset of clients provides privatized binary evaluations of model performance. This mechanism preserves correlation structure and achieves higher utility by more effectively leveraging the underlying relationship between features and responses.
2. When to transfer. We quantify the transferability, i.e., the utility of an LDP-perturbed dataset, to assess whether a source is beneficial or detrimental. Our proposed LDP-tailored evaluation process provides an unbiased estimate of this utility, enabling more effective identification of negative datasets or classifiers.
3. How to transfer. We introduce two new techniques, guided by our utility evaluation, to improve performance of classifiers obtained under LDP:
  - Model Reversal (MR): When a noised classifier performs worse than random guessing, we reverse its decision boundary. This effectively corrects negative datasets without discarding them entirely.
  - Model Averaging (MA): We adapt ensemble-learning ideas to the LDP context, assigning weight to each reversed classifier based on its estimated utility. The resulting averaged classifier tends to outperform any single classifier, especially in the presence of high noise.

We address key challenges in private learning under LDP by introducing a novel framework inspired by transfer learning. Our main contributions are summarized below:

1. **Linking LDP and Transfer Learning.** We recast private learning under LDP as a transfer learning problem, interpreting the noisy dataset as a source and the true data as a target. Building on Qin et al. (2024), we use their proposed definition of transferability to measure dataset utility under a given privacy budget.
2. **Techniques for Private Learning.** We develop three LDP-tailored tools:
  - An evaluation scheme that requests privatized binary feedback on a classifier’s performance, based on which we provide unbiased accuracy estimates, effectively addressing the lack of target data under LDP.
  - Model reversal, which salvages weak classifiers with below-50% accuracy by inverting their decision boundaries, effectively leveraging negative datasets.
  - Model averaging, which averages multiple reversed weak classifiers, weighting them by their estimated utility under LDP.
3. **Theoretical Guarantees.** We provide excess risk bounds for our proposed methods, illustrating how noise affects classification performance under LDP and demonstrating how MR and MA help reduce this bound.

Beyond these contributions, our proposed techniques are broadly applicable across various data structures and classification algorithms. The framework can be easily adapted to different privacy protection mechanisms by modifying the noise injection strategy. As a demonstration, we present a functional data classification example that, to our knowledge, represents the first LDP-based approach for modeling functional covariates.

In the following sections, we formalize these ideas and demonstrate their effectiveness both theoretically and empirically. Section 2 reviews related work. Section 3 introduces our utility evaluation process and the proposed techniques tailored for the LDP setting. Section 4 establishes excess risk bounds for the proposed classifiers. Section 5 presents the complete process of building a functional classifier under LDP using our framework. Section 6 reports experimental results that demonstrate the effectiveness of the proposed techniques. Section 7 presents real-data applications, and Section 8 concludes the paper.

## 2. Related work

In this section, we review the literature on supervised learning under LDP, with a particular focus on classification methods. We then discuss key concepts from transfer learning that motivate our approach, followed by a brief overview of general classification techniques. Finally, we highlight relevant developments in functional data analysis that inform our application setting.

### Supervised Learning Under LDP.

Given challenges arising from data correlations and high dimensionality, existing research on supervised learning under LDP is limited. Although a few studies have explored learning under LDP, preserving both privacy and utility remains a significant challenge. Some research focuses on empirical risk minimization (ERM), treating the learning process as an optimization problem solved through defining a series of objective functions. Wang et al. (2019a) constructed a class of machine learning models under LDP that can be expressed as ERM, and solved by stochastic gradient descent (SGD). To address the high dimensionality issue, Liu et al. (2020) privately selected the top-k dimensions according to their contributions in each iteration of federated SGD. Deep learning models under LDP have also been studied; for example, Sun et al. (2021) employed adaptive data perturbation and parameter shuffling to mitigate privacy loss. Nevertheless, balancing privacy guarantees with competitive performance is still an open problem, particularly in large-scale, high-dimensional settings.

### Classification Under LDP.

Currently, only a few classification algorithms have been developed under LDP. Among the most relevant are the works of Yilmaz et al. (2020); Berrett and Butucea (2019); Ma and Yang (2024). Yilmaz et al. (2020) proposed a naive Bayes classifier using LDP-based frequency and mean estimators. Their method assumes independence among features, which may limit its applicability in real-world settings. While they suggest dimensionality reduction techniques such as principal component analysis and discriminant component analysis for high-dimensional data, they do not detail how these can be implemented in the LDP context, where raw data are not directly accessible. Berrett and Butucea (2019) introduced a histogram-based classifier and established minimax rates for excess risk. Their approach applies Laplace noise with a scale parameter that grows exponentially with the number of features, potentially introducing substantial distortion, even in moderately low-dimensional settings, which may severely degrade classification performance. Ma and Yang (2024) considered the setting where additional public data are available and proposed an LDP decision tree classifier that achieves minimax-optimal rates under this setup. As discussed in Section 1, leveraging public data effectively transforms the problem into a transfer learning task, aligning with the framework in Cai and Wei (2021). Their algorithm also remains implementable in the absence of public data, in which case the procedure reduces to a standard locally private tree classifier.

### Transfer Learning.

Transfer learning (Torrey and Shavlik, 2010) enhances a target task by leveraging knowledge acquired from related source tasks. Its success depends on accurately quantifying the relationship between source and target domains to assess how informative the source is and to detect negative sources. Negative transfer occurs when a poorly matched source degrades target performance (Weiss et al., 2016). The identification of informative or negative sources critically depends on the chosen metric for transferability. In a series of parameter-based transfer learning works, it is common to measure the transferability of a source dataset by quantifying the distance between the parameters of interest in the source and target datasets; see Li et al. (2022); Lin and Reimherr (2022); Tian and Feng (2022). Yet, parameter-based measures alone can be misleading in classification settings, as smaller parameter distances do not necessarily imply higher transferability (Qin et al., 2024). Alternative approaches assess the distributional similarity through a relative signal exponent (Cai and Wei, 2021) or label-matching probabilities (Qin et al., 2024). In the context of LDP, available noised datasets, which serve as source data, are often prone to negative transfer because severe perturbations can distort key information and hinder reliable transferability assessment.

### Standard Classification Methods.

Classification tasks appear across numerous real-world scenarios, ranging from medical diagnoses to spam detection. Classic methods include discriminant analysis (Hall et al., 2001), Naïve Bayes classification (Dai et al., 2017), and logistic regression (Leng and Müller, 2006), while contemporary research often employs more advanced approaches such as support vector machines (SVM, Blanchard et al., 2008) or neural-network-based classifier (Saravanan and Sasithra, 2014). Comprehensive overviews of classification methods can be found in Kotsiantis et al. (2007); Kumari and Srivastava (2017). From a theoretical perspective, many works investigate excess risk bounds to establish statistical guarantees. For instance, Blanchard et al. (2008) derived non-asymptotic bounds for SVM-based classifiers, Sang et al. (2022) developed similar results for distance-weighted discrimination (DWD), and Ko et al. (2023) studied the excess risk convergence rates of neural network-based classifiers. In transfer learning contexts, Cai and Wei (2021) and Qin et al. (2024) analyzed the excess risk of transfer-learning-based classifiers. However, results on classification under LDP remain limited, particularly in understanding how noise injection impacts both model training and theoretical guarantees.

### Functional Data Analysis.

Functional data, such as curves or surfaces defined over continuous domains, are inherently infinite-dimensional (Ramsay and Silverman, 2005; Horváth and Kokoszka, 2012). Common examples include time-continuous measurements collected by wearable devices, which are often associated with sensitive health information. To facilitate statistical analysis, recent research has focused on functional projection techniques that map functional observations into finite-dimensional representations (Horváth and Rice, 2015; Pomann et al., 2016; Kraus and Stefanucci, 2019; Bai et al., 2023). Several differential privacy methods have been developed for functional data (Hall et al., 2013; Mirshani et al., 2019; Jiang et al., 2023; Lin and Reimherr, 2023), but most are based on the global DP setting, where a trusted data curator has access to the entire dataset. In contrast, LDP requires that data be perturbed at the individual level prior to collection, introducing unique challenges for functional data. As a result, training predictive models on functional data under LDP remains an open and underexplored problem, leaving a gap in privacy-preserving techniques for infinite-dimensional data.

## 3. Privacy-Preserving Classification under LDP

Under local differential privacy, observed data are perturbed to protect sensitive information, which often leads to a loss in utility. In this section, we introduce a utility measure to evaluate the quality of perturbed data and propose novel techniques to improve classifier performance under LDP without compromising privacy.

### 3.1 Data and Notation

In practice, original data collected from clients can be of diverse forms, including high-dimensional data (Araveeporn, 2021), functional data (Kraus and Stefanucci, 2019), images (Rawat and Wang, 2017), and text (Blitzer et al., 2006). To derive usable features from these data under privacy constraints, one often applies suitable representation methods and then adds random noise to the resulting features or labels. In this subsection, we detail the representation process and introduce the notations used throughout.

Let 𝒳 be an instance set, i.e., the space of original inputs (e.g., the diverse data types mentioned above). Let 𝒵 be a feature space, with $𝒵=R^{d}$ as a common example, where elements in 𝒵 correspond to finite-dimensional representations of the original input. Let 𝒴={-1,1} denote the label set for binary classification. A mapping function is characterized by a distribution over 𝒳 and a (stochastic) mapping function ζ:𝒳→𝒴. For an instance X∈𝒳, the value ζ(X) corresponds to the probability that its label Y is 1. Let Q denote the joint distribution of {X,Y}, and let $Q_{X}$ be the marginal distribution of X. We write $Q_{Y|X}(x)=\zeta (x)$ to represent the conditional distribution P(Y=1∣X=x).

A representation function ℛ:𝒳→𝒵 maps instances to features, thereby inducing a distribution over 𝒵 and a (stochastic) mapping function η:𝒵→𝒴. Specifically, for any Borel set B⊆𝒵 such that ${ℛ}^{-1}(B)$ is $Q_{X}$-measurable,

$$P\left(Z\in B\right)=Q_{X}\left({ℛ}^{-1}\left(B\right)\right).$$

Let P denote the joint distribution of {Z,Y}, and let $P_{Z}$ be the marginal distribution of Z. We denote by η(z)≜P(Y=1∣Z=z) the conditional distribution of Y given Z, where

$$\eta (Z)=E_{Q_{X}}[\zeta (X)|ℛ(X)=Z].$$

Note that η(Z) may be a stochastic function even if ζ(X) is not. This is because the function ℛ can map two instances with different labels to the same feature representation (Ben-David et al., 2006).

Next, consider an ε-LDP mechanism that transforms (Z,Y) into $\left(Z^{\left(\varepsilon_{z}\right)},Y^{\left(\varepsilon_{y}\right)}\right)$, where $\varepsilon =\left(\varepsilon_{z},\varepsilon_{y}\right)$ denotes the privacy budget allocation with $\varepsilon_{z},\varepsilon_{y}>0$. When both Z and Y require protection, the budget is split such that $\varepsilon_{z}+\varepsilon_{y}=\varepsilon$. Notable cases include protecting only covariates ($\varepsilon_{z}=\varepsilon ,\varepsilon_{y}=\infty$, meaning no privacy constraint on Y), or only labels $\left(\varepsilon_{y}=\varepsilon ,\varepsilon_{z}=\infty \right)$, where the summation constraint does not apply as the entire budget is assigned to one component. One way to achieve ε-LDP is by setting:

(1) $$Z^{\left(\varepsilon_{z}\right)}=Z+\delta^{\left(\varepsilon_{z}\right)},\;P\left(Y^{\left(\varepsilon_{y}\right)}=Y\right)=q^{\left(\varepsilon_{y}\right)},$$

where the noise $\delta^{\left(\varepsilon_{z}\right)}$ follows a distribution $D^{\left(\varepsilon_{z}\right)}$ whose scale is calibrated to the sensitivity of Z under the chosen $\varepsilon_{z}$-LDP mechanism, and smaller $\varepsilon_{z}$ corresponds to a larger noise scale. For example, if $Z\in [a,b{]}^{d}$, then under the Laplace mechanism (Dwork et al., 2006) one can add i.i.d. Laplace $\left(0,\lambda_{z}\right)$ noise to each coordinate with $\lambda_{z}=d(b-a)/\varepsilon_{z}$. A common choice for $q^{\left(\varepsilon_{y}\right)}$ is $e^{\varepsilon_{y}}/\left(1+e^{\varepsilon_{y}}\right)\in [1/2,1)$, corresponding to the classical randomized response mechanism (Warner, 1965). This added noise weakens the dependency between Z and Y, thereby degrading data utility. In Section 3.2, we show how utility is affected by the allocation of the privacy budget.

Parallel to the original distribution P, let $P^{\left(\varepsilon \right)}$ be the joint distribution of $\left(Z^{\left(\varepsilon_{z}\right)},Y^{\left(\varepsilon_{y}\right)}\right)$. We denote its marginal distribution by $P_{Z}^{\left(\varepsilon \right)}$ and the conditional distribution by $P_{Y|Z}^{\left(\varepsilon \right)}(z)=P\left(Y^{\left(\varepsilon_{y}\right)}=1|Z^{\left(\varepsilon_{z}\right)}=z\right)≔\eta^{\left(\varepsilon \right)}(z)$. Under LDP, we only observe the noised data $\left(Z^{\left(\varepsilon_{z}\right)},Y^{\left(\varepsilon_{y}\right)}\right)$, which defines the source distribution $P^{\left(\varepsilon \right)}$. Our goal, however, is to learn a model that performs well on the unobserved target distribution P (and ultimately Q), in order to gain insights into the real world. This discrepancy between the source and target distributions highlights a fundamental challenge in the LDP setting.

In this work, we focus on developing techniques that improve classification performance under a given LDP mechanism. The methods proposed in the following sections can be adapted to different variants of LDP (Qin et al., 2023) by appropriately modifying the noise injection procedure in Equation (1). For concreteness, we use the standard ε-LDP framework to illustrate our approach throughout this paper.

### 3.2 Measure of Utility

In this subsection, we introduce a utility measure for the perturbed dataset $\left(Z^{\left(\varepsilon_{z}\right)},Y^{\left(\varepsilon_{y}\right)}\right)$ by adopting the concept of dataset transferability proposed by Qin et al. (2024). For any feature point $z_{0}\in 𝒵$, we define the pointwise utility function using two independent draws: (Y,Z)~P from the original distribution and $\left(Y^{\left(\varepsilon_{y}\right)},Z^{\left(\varepsilon_{z}\right)}\right)\sim P^{\left(\varepsilon \right)}$ from the privatized distribution. Specifically,

(2) $$g^{\left(\varepsilon \right)}\left(z_{0}\right)=P\left(Y^{\left(\varepsilon_{y}\right)}=Y|Z=z_{0},\;Z^{\left(\varepsilon_{z}\right)}=z_{0}\right)\in \left[0,1\right],$$

which represents the probability that the label from the original distribution with $Z=z_{0}$ agrees with the label from the privatized distribution with $Z^{\left(\varepsilon_{z}\right)}=z_{0}$. Proposition 2 below clarifies how $g^{\left(\varepsilon \right)}\left(z_{0}\right)$ relates to the mapping functions η and $\eta^{\left(\varepsilon \right)}$, and how it is affected by the privacy budget allocation $\left(\varepsilon_{z},\varepsilon_{y}\right)$.

**Proposition 2** *With the mapping functions* η *and* $\eta^{\left(\varepsilon \right)}$ *defined above, we have*

(3) $$g^{\left(\varepsilon \right)}\left(z_{0}\right)=2\left(\eta \left(z_{0}\right)-\frac{1}{2}\right)\left(\eta^{\left(\varepsilon \right)}\left(z_{0}\right)-\frac{1}{2}\right)+\frac{1}{2},$$

*where*

(4) $$\eta^{\left(\varepsilon \right)}\left(z_{0}\right)-\frac{1}{2}=2\left(q^{\left(\varepsilon_{y}\right)}-\frac{1}{2}\right)E_{P_{Z}}\left[\omega \left(z|z_{0},\varepsilon_{z}\right)\left(\eta (z)-\frac{1}{2}\right)\right]$$

*and*

(5) $$\omega \left(z|z_{0},\varepsilon_{z}\right)=\frac{D^{\left(\varepsilon_{z}\right)}\left(\delta_{z}^{\left(\varepsilon_{z}\right)}=z_{0}-z\right)}{E_{P_{Z}}\left\{D^{\left(\varepsilon_{z}\right)}\left(\delta_{z}^{\left(\varepsilon_{z}\right)}=z_{0}-z\right)\right\}}.$$

**Figure 1:**
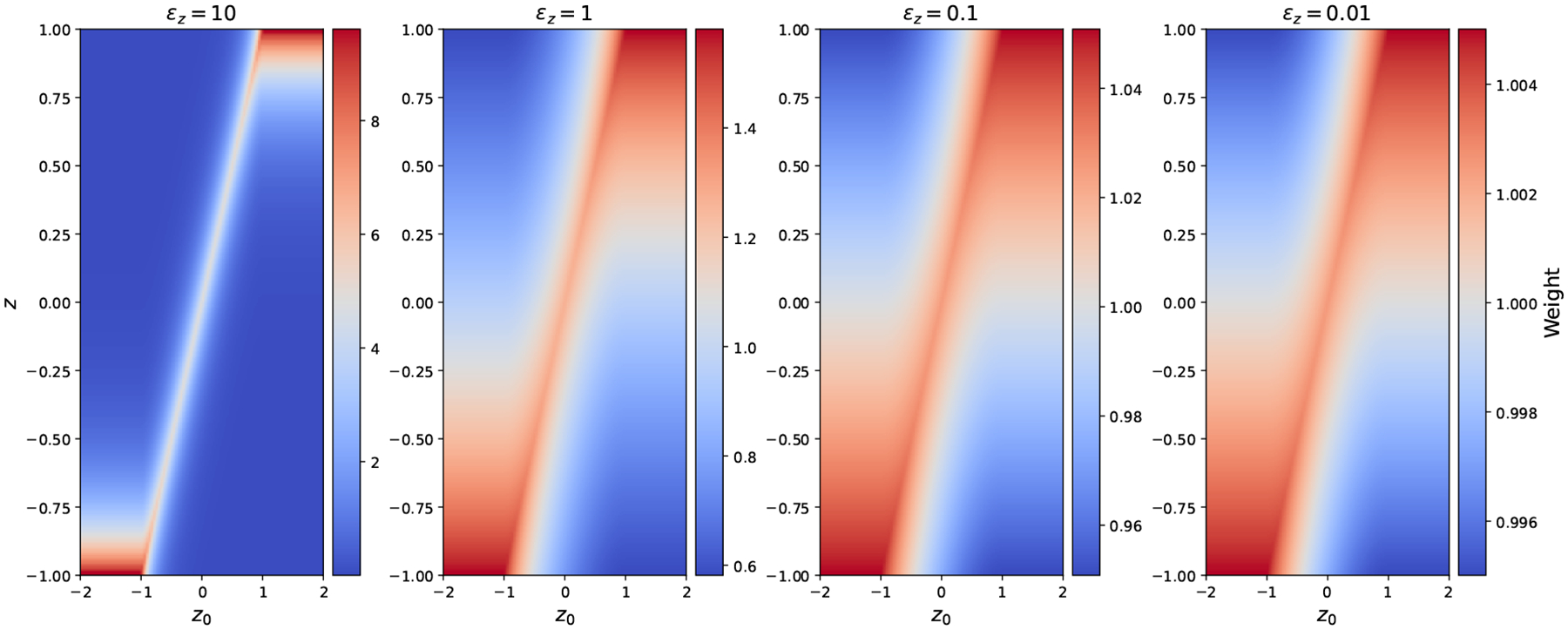
Heatmaps of the weight function $\omega \left(z|z_{0},\varepsilon_{z}\right)$ changing with z for various values of $z_{0}$ and $\varepsilon_{z}$, where we assume z~U(-1,1) and $z_{0}=z+\delta_{z}^{\left(\varepsilon_{z}\right)}$ with $\delta_{z}^{\left(\varepsilon_{z}\right)}$ drawn from a Laplace distribution having mean zero and scale $2/\varepsilon_{z}$. As $\varepsilon_{z}$ decreases, the value intervals of the color bars become increasingly concentrated around 1.

In Proposition 2, $\omega \left(z|z_{0},\varepsilon_{z}\right)$ is analogous to a continuous weighting scheme, satisfying $E_{P_{Z}}\left(\omega \left(z|z_{0},\varepsilon_{z}\right)\right)=1$. As discussed in Qin et al. (2024), Equation (3) indicates that $g^{\left(\varepsilon \right)}\left(z_{0}\right)$ depends on how η and $\eta^{\left(\varepsilon \right)}$ deviate from 1/2. When they deviate in the same direction, $g^{\left(\varepsilon \right)}\left(z_{0}\right)\geq 1/2$, making the noised dataset informative; otherwise, it can be negative. Equation (4) illustrates how the privacy budget parameters ${(\varepsilon }_{z},\varepsilon_{y})$ jointly influence $g^{\left(\varepsilon \right)}\left(z_{0}\right)$. To gain clearer insight into their roles, we consider the following two notable cases:

1. Protecting only Y: Let $\varepsilon_{y}=\varepsilon$ and $\varepsilon_{z}=\infty$. We then have $P_{Z}^{\left(\varepsilon \right)}=P_{Z}$ but $P_{Y|Z}^{\left(\varepsilon \right)}\neq P_{Y|Z}$, which matches the classic posterior drift scenario (Qin et al., 2024). In this setting,
$$\eta^{\left(\varepsilon \right)}\left(z_{0}\right)-\frac{1}{2}=2\left(q^{\left(\varepsilon_{y}\right)}-\frac{1}{2}\right)\left(\eta \left(z_{0}\right)-\frac{1}{2}\right),$$
  and the dataset remains informative when $q^{\left(\varepsilon_{y}\right)}\geq 1/2$, meaning the perturbed data and the original data share the same Bayes classifier; otherwise, it becomes negative.
2. Protecting only Z: Let $\varepsilon_{z}=\varepsilon$ and $q^{\left(\varepsilon_{y}\right)}=1$. Then $\eta^{\left(\varepsilon \right)}\left(z_{0}\right)-1/2$ primarily depends on the weight $\omega \left(z|z_{0},\varepsilon_{z}\right)$ defined in Equation (5). To examine the impact of $\varepsilon_{z}$ on this weight, we consider a special case where z~U(-1,1), the uniform distribution on the interval (−1, 1). The noise $\delta_{z}^{\left(\varepsilon_{z}\right)}$ is drawn from a Laplace distribution with mean zero and scale parameter $2/\varepsilon_{z}$, which satisfies ε-LDP. We then compute the weight for $z_{0}\in [-2,2]$ with $\varepsilon_{z}\in \{10,1,0.1,0.01\}$. Figure 1 illustrates how the weight function $\omega \left(z|z_{0},\varepsilon_{z}\right)$ varies with z for different $z_{0}$ and $\varepsilon_{z}$, where the range of weight becomes increasingly concentrated around 1 as $\varepsilon_{z}$ approaches 0. Given that $\int_{z}\omega (z|\left.z_{0},\varepsilon_{z}\right)dz=1$, these heatmaps indicate that as $\varepsilon_{z}$ decreases (i.e., stronger privacy constraints and more noise), the weight function becomes highly diffuse, spreading nearly uniformly over z∈[-1,1] rather than concentrated around the diagonal $z=z_{0}$ for $z_{0}\in [-1,1]$ or at the boundaries z=-1 and z=1 when $z_{0}<-1$ and $z_{0}>1$, respectively. Consequently, as privacy constraints tighten and the weight function grows more diffuse, the deviation between $\eta^{\left(\varepsilon \right)}(z)-\frac{1}{2}$ and $\eta (z)-\frac{1}{2}$ becomes larger.

### 3.3 Evaluation of Utility

Utility assessment is crucial when integrating information from multiple data sources. Unlike in transfer learning, where one can directly evaluate source-trained models on target data, the LDP setting lacks access to unperturbed target data. To address this, we propose a novel alternative approach that requires only a noised binary evaluation from each client. Based on these privatized responses, we provide an unbiased estimate of the utility of a model trained on perturbed data.

Given a perturbed dataset ${𝒟}_{0}={\left\{\left(z_{i}^{\left(\varepsilon_{z}\right)},y_{i}^{\left(\varepsilon_{y}\right)}\right)\right\}}_{i=1}^{n_{0}}$, our goal is to estimate its utility $g^{\left(\varepsilon \right)}\left(z_{0}\right)$, as defined in Equation (2), for any given $z_{0}$ drawn from the distribution $P_{Z}$. A key challenge is that both $y\left(z_{0}\right)$ and $y^{\left(\varepsilon_{y}\right)}\left(z_{0}\right)$ are unobservable. To overcome this, instead of requesting noisy feature-label pairs from each client, we apply the classifier $f^{\left(\varepsilon \right)}\left(z_{0}\right)$, trained on ${𝒟}_{0}$, to each client in evaluation set and request a privatized binary response indicating whether $y\left(z_{0}\right)={\overset{ˆ}{y}}^{\left(\varepsilon_{y}\right)}\left(z_{0}\right)$, where ${\overset{ˆ}{y}}^{\left(\varepsilon_{y}\right)}\left(z_{0}\right)=\text{sign}\left(f^{\left(\varepsilon \right)}\left(z_{0}\right)\right)$. This approach enables a more efficient form of querying, as it preserves the correlation between the response and the functional covariate in the target distribution while injecting significantly less noise, since only a single binary response is privatized per client. Furthermore, measuring the utility of ${𝒟}_{0}$ in this way is equivalent to estimating the classification accuracy of $f^{\left(\varepsilon \right)}\left(z_{0}\right)$ trained on it. This interpretation aligns with Proposition 2, which indicates that higher dataset utility corresponds to better Bayes classification performance achievable from that dataset.

In what follows, we present the detailed procedures for classifier training and dataset utility evaluation. These involve two distinct types of client-side information access: collecting noised observation pairs and obtaining privatized binary evaluations. We assume a total of N clients, partitioned into a training set ${𝒟}_{\text{train}}$ of size $N_{0}$ and an evaluation set ${𝒟}_{\text{eval}}$ of size $N_{1}$, where $N_{0}+N_{1}=N$.

1. **Classifier Training.** The server collects perturbed data ${\left\{\left(z_{i}^{\left(\varepsilon_{z}\right)},y_{i}^{\left(\varepsilon_{y}\right)}\right)\right\}}_{i\in {𝒟}_{\text{train}}}$ from the training clients to fit or construct multiple weak classifiers. Specifically, for each b=1,…,B, we randomly sample $n_{0}\left(<N_{0}\right)$ pairs from ${𝒟}_{\text{train}}$, denoted as ${𝒟}_{\text{train}}^{\left(b\right)}$. Using these samples, we construct B weak classifiers ${\left\{f^{\left(b\right)}\right\}}_{b=1}^{B}$. (Here and after, we ignore the ε in $f^{\left(b\right)}$ for simple representation.) Each client only uploads once perturbed observation, thus preserving their privacy guarantee.
2. **Utility Evaluation.** For the $N_{1}$ clients in the evaluation set, we do not collect their perturbed observations. Instead, each evaluation client provides a noised binary indicator of misclassification, which is used to evaluate a classifier’s performance. Specifically, we split the evaluation set into B subsets: ${𝒟}_{\text{eval}}^{\left(b\right)},b=1,\ldots ,B$. For a client $i\in {𝒟}_{\text{eval}}^{\left(b\right)}$ with true observation ${(x}_{i},y_{i})$, we compute
(6) $${\overset{ˆ}{y}}_{i}≔I\left[f^{\left(b\right)}\left\{ℛ\left(x_{i}\right)\right\}>0\right]\;\text{and}\;r_{i}≔I\left({\overset{ˆ}{y}}_{i}=y_{i}\right).$$
  Each client then reports a privatized value $r_{i}^{\prime }$ using randomized response, where
(7) $$P\left(r_{i}^{\prime }=r_{i}\right)=q=\frac{e^{\varepsilon_{v}}}{1+e^{\varepsilon_{v}}}\;\text{and}\;P\left(r_{i}^{\prime }\neq r_{i}\right)=1-q.$$
  Theorem 3 shows that this evaluation mechanism satisfies ε-LDP when $\varepsilon_{v}=\varepsilon$, and provides an unbiased estimate of the accuracy of the classifier $f^{\left(b\right)}$. This estimate can be taken as an evaluation of the utility of the dataset ${𝒟}_{\text{train}}^{\left(b\right)}$, for b=1,…,B.

**Theorem 3** *Let* ℳ *be the privacy mechanism mapping* (x,y) *to* $r^{\prime }$ *as described above. Then* ℳ *satisfies* $\varepsilon_{v}$-*LDP. Furthermore, let* $r^{\left(b\right)}$ *be the true classification accuracy of* $f^{\left(b\right)}$ *under the target distribution* P, *and let* $n_{1}^{\left(b\right)}=\left|{𝒟}_{\text{eval}}^{\left(b\right)}\right|$. *Define*

(8) $${\tilde{r}}^{\left(b\right)}=\frac{{\overset{ˆ}{r}}^{\left(b\right)}+q-1}{2q-1},\;\text{where}\;{\overset{ˆ}{r}}^{\left(b\right)}=\frac{\sum_{i\in {𝒟}_{\text{eval}}^{\left(b\right)}}r_{i}^{\prime }}{n_{1}^{\left(b\right)}}.$$

*Then* $E\left({\tilde{r}}^{\left(b\right)}\right)=r^{\left(b\right)}$, *and* $\text{Var}\left({\tilde{r}}^{\left(b\right)}\right)\leq {\left(\frac{e^{\varepsilon_{v}}+1}{e^{\varepsilon_{v}}-1}\right)}^{2}{\left(4n_{1}^{\left(b\right)}\right)}^{-1}$.

Theorem 3 indicates that as $n_{1}^{\left(b\right)}$ increases, ${\tilde{r}}^{\left(b\right)}$ converges more closely to the true classification accuracy of $f^{\left(b\right)}$ under LDP. Consequently, enlarging the evaluation set substantially enhances the precision of accuracy estimates and assists in selecting the better-performing weak classifiers. Since heavily perturbed data may limit the gains from training on more samples, these findings suggest allocating a greater proportion of clients to evaluation rather than training. Simulation results in Appendix A.3 provide evidence supporting this strategy. In addition, during the evaluation step, each client in ${𝒟}_{\text{eval}}^{\left(b\right)}$ evaluates only a single classifier, ensuring that their privacy budget remains ε by setting $\varepsilon_{v}=\varepsilon$. If a client were to evaluate multiple classifiers, the budget would need to be divided among them, potentially reducing the precision of each evaluation. Specifically, when evaluating k classifiers, the privacy budget must be allocated as $\varepsilon_{v}=\varepsilon /k$ for each evaluation, leading to an increase in $\text{Var}\left({\tilde{r}}^{\left(b\right)}\right)$ as k grows.

### 3.4 Model Reversal and Model Averaging

Under LDP, the added noise can be substantial, causing the weak classifiers ${\left\{f^{\left(b\right)}\right\}}_{b=1}^{B}$ to perform poorly. As shown in Proposition 2, it is possible for $g^{\left(\varepsilon \right)}(z)$ to fall below 1/2, indicating a negative dataset that performs worse than random guessing. We propose model reversal and model averaging to extract useful information from such seemingly uninformative data and improve classification performance. The implementation of these techniques builds on the unbiased utility evaluation established in Theorem 3.

#### Model Reversal.

We first propose a novel procedure to enhance the performance of each weak classifier. Formally, for a weak classifier $f^{\left(b\right)}$, we define its reversed classifier as

(9) $$f^{*(b)}=\left\{\begin{array}{ll} -f^{\left(b\right)}, & \text{if}\;\text{its}\;\text{estimated}\;\text{accuracy}\;{\tilde{r}}^{\left(b\right)}<0.5,\\ f^{\left(b\right)}, & \text{otherwise.} \end{array}\right.$$

The model reversal process defined in Equation (9) means that for a given classifier $f^{\left(b\right)}$, its sign is reversed if its estimated accuracy ${\tilde{r}}^{\left(b\right)}<0.5$; otherwise, it remains unchanged. Consequently, the estimated accuracy of the reversed classifier $f^{*(b)}$ becomes ${\tilde{r}}^{*(b)}=\text{max}\left\{{\tilde{r}}^{\left(b\right)},1-\right.\left.{\tilde{r}}^{\left(b\right)}\right\}$, ensuring it is larger than 0.5. This idea exploits the fact that classification primarily depends on the direction of the coefficient vector relative to the decision boundary, rather than its magnitude. By identifying whether a classifier is less than random guessing, we can flip its decision boundary to ensure it surpasses 50% accuracy. Here we focus on binary classification, where model reversal reduces to a simple “flip or keep” decision. Extending the MR idea to multi-class problems is an interesting direction for future work. For example, one could reduce a K-class problem to K one-vs-rest binary subproblems, or apply permutation-based corrections to the predicted class labels of each weak classifier, guided by confusion matrices estimated from suitably designed LDP-protected queries in the evaluation step.

#### Model Averaging.

Next, given B reversed weak classifiers ${\left\{f^{*(b)}\right\}}_{b=1}^{B}$ with estimated accuracies ${\left\{{\tilde{r}}^{*(b)}\right\}}_{b=1}^{B}$, we combine them by weighted averaging:

(10) $$w_{b}=\frac{\text{max}\left\{{\tilde{r}}^{*(b)}-r_{0},0\right\}}{\sum_{j=1}^{B}\;\text{max}\left\{{\tilde{r}}^{*(j)}-r_{0},0\right\}},$$

where $r_{0}\in (0.5,1)$ is a server-specified cutoff. Classifiers with ${\tilde{r}}^{*(b)}\leq r_{0}$ receive weight 0, and the final averaged classifier is

(11) $$f^{†}\left(\cdot \right)=\sum_{b=1}^{B}w_{b}f^{*\left(b\right)}\left(\cdot \right).$$

In Equation (11), we aggregate reversed weak classifiers by a weighted average. For parametric classifiers where $f^{*(b)}(\cdot )$ is linear in the model parameters, Equation (11) is equivalent to averaging the corresponding parameter vectors. More generally, when the weak classifiers are nonlinear in their parameters or do not share a common parameterization, the same utility-based weights $\left\{w_{b}\right\}$ from Equation (10) can be combined with alternative aggregation rules, such as weighted voting or averaging predicted probabilities or decision scores.

The effectiveness of MR and MA relies on accurate utility estimation. Theorems 5 and 6 show that these techniques improve excess risk bounds. Our experiments further confirm that MR and MA significantly enhance classification accuracy.

### 3.5 Single-Server Classification with MRMA

This subsection integrates the previously introduced components, privacy-preserving data collection, utility evaluation, model reversal, and model averaging, into a unified procedure for classifier construction on a single server. The full process is presented in Algorithm 1, which takes as input the randomized mechanism, privacy budget, and learning parameters, and returns a final classifier trained under LDP.

**Algorithm 1:** Single-Server Classification with MRMA

1:	**procedure** $\text{Server}\left(ℛ,N_{0},N_{1},n_{0},B,\varepsilon ,\varepsilon_{z},\varepsilon_{y},r_{0}\right)$	
2:	Divide clients into a training set ${𝒟}_{\text{train}}$ and an evaluation set ${𝒟}_{\text{eval}}$ with $\left|{𝒟}_{\text{train}}\right|=N_{0},\left|{𝒟}_{\text{eval}}\right|=N_{1}$ and spilt the evaluation set into B subsets,${\left\{{𝒟}_{\text{eval}}^{\left(b\right)}\right\}}_{b=1}^{B}$.	
3:	**for** each client in the training set **do**	
4:	Obtain $\left(z_{i}^{\left(\varepsilon_{z}\right)},y_{i}^{\left(\varepsilon_{y}\right)}\right)$ by Equation (1).	▷ privacy-preserving data collection
5:	**end for**	
6:	**for b=1,…,B do**	
7:	Randomly draw $n_{0}$ samples from ${𝒟}_{\text{train}}$ without replacement and denote as ${𝒟}_{\text{train}}^{\left(b\right)}$.	
8:	Build a classifier $f^{\left(b\right)}$ based on ${\left\{\left(z_{i}^{\left(\varepsilon_{z}\right)},y_{i}^{\left(\varepsilon_{y}\right)}\right)\right\}}_{i\in {𝒟}_{\text{train}}^{\left(b\right)}}$.	▷ server-specified classifier
9:	**for** each client in the evaluation set ${𝒟}_{\text{eval}}^{\left(b\right)}$ **do**	
10:	Compute $r_{i}^{\prime }$ by Equations (6) and (7).	
11:	**end for**	
12:	Estimate ${\tilde{r}}^{\left(b\right)}$ by Equation (8).	▷ utility evaluation
13:	Compute $f^{*(b)}$ by Equation (9).	▷ model reversal
14:	**end for**	
15:	Estimate $w={\left(w_{1},\ldots ,w_{B}\right)}^{⊤}$ by Equation (10) with cutoff value $r_{0}$.	
16:	**return** The final estimated classifier $f^{†}$ by Equation (11).	▷ model averaging
17:	**end procedure**	

#### Sample Size Balancing.

In practice, when the total sample size N is large, increasing the training size $N_{0}$, the evaluation size $N_{1}$, and the number of classifiers B can improve performance. However, for a fixed N, it is important to carefully choose $N_{0},N_{1}$ and B. Larger values of $N_{0}$ and B help train more effective weak classifiers, while smaller values ensure sufficient data in each evaluation subset. Section 4 provides theoretical guidance on setting these parameters. We also empirically study their impact in Appendix A.2 and A.3, offering practical recommendations.

#### Broad Applicability.

While this work focuses on a specific algorithmic structure, the proposed evaluation process and MRMA techniques are broadly applicable. Regarding data types, our framework accommodates vectors, functional data, images, or text, depending on the representation function and classifier specified at line 8 in Algorithm 1. Regarding privacy mechanisms, MRMA is compatible with various LDP variants by adjusting how noise is added in the training and evaluation phases, as implemented in lines 4 and 10 of Algorithm 1, respectively. This flexibility enables the method to adapt to a wide range of practical applications with different data modalities and privacy requirements.

#### Online data.

Although our primary focus is on protecting static information such as individual profiles, many real-world systems produce continuous data streams from sensors or digital interactions. Our proposed procedure naturally extends to such online settings. On one hand, accuracy estimates for existing weak classifiers can be updated incrementally, allowing dynamic adjustment of their weights in the aggregated model. On the other hand, if performance deteriorates on new data, additional weak classifiers can be trained on newly collected, privatized samples. As a result, the final classifier can adapt over time by incorporating fresh models and reweighting existing ones, effectively tracking changes in the underlying data distribution.

### 3.6 Multi-Server Classification with MRMA under Heterogeneity

In many real-world scenarios, data are distributed across multiple servers. However, these servers may exhibit varying degrees of heterogeneity in their local data, which existing methods do not always handle effectively. Our proposed techniques extend naturally to the multi-server setting under LDP. Assume there are K servers, each with a locally trained classifier $f_{k}(x)$. These servers share their models with one another. Each server can then re-evaluate the shared classifiers and apply MRMA, as described in Sections 3.3 and 3.4, treating classifiers from different servers as a collection of weak classifiers. Details of model evaluation and MRMA in this setting are provided in the following two paragraphs.

#### Perturbation and Evaluation.

A server with sufficient clients can split its evaluation set into B+K parts, using B parts for evaluating its internal weak classifiers and K parts for evaluating the externally received classifiers ${\left\{f_{k}\right\}}_{k=1}^{K}$. Each evaluation is performed via randomized response at privacy level ε, as in Section 3.3. When data are limited, one can sequentially evaluate internal and external classifiers, dividing the privacy budget accordingly (by splitting ε appropriately).

#### MRMA.

Upon receiving performance assessments for all K classifiers from multiple servers, each server can independently apply MR and MA with a cutoff $r_{0}^{*}$ (see Equation (10) in Section 3.4) and obtain the final classifier $f_{k}^{†}$ for the k-th server. Because the servers and their data distributions may be highly heterogeneous, employing our MRMA approach can mitigate negative transfer effects by assigning small or zero weights to less relevant models. Overall, by integrating LDP with MR and MA, each server can capitalize on the shared knowledge from others, achieving improved classification performance while still honoring the local privacy constraints.

Our discussion of the multi-server setting focuses on how locally trained ε-LDP classifiers can be re-evaluated and then combined via MRMA to improve each server’s final decision rule. In practice, distributed learning across multiple servers can involve additional considerations that are widely studied in the federated learning literature (Truex et al., 2020; Sun et al., 2021). Our evaluation and MRMA procedures can be used in such settings, as they only require a collection of candidate ε-LDP classifiers and their utility estimates.

## 4. Theoretical Guarantees for Classification under LDP

In this section, we analyze how the LDP mechanism affects classification performance and demonstrate the effectiveness of our proposed techniques through their improvements in excess risk bounds. We begin with necessary definitions for Bayes classifiers and proceed to establish our theoretical guarantees.

Recall that η(z) denotes the conditional probability function, and the corresponding Bayes classifier under the original (unperturbed) distribution P is given by $h^{*}(z)=\text{sign}\{\eta (z)\geq 1/2\}$. The expected classification accuracy of $h^{*}$ is $\frac{1}{2}+E_{P_{Z}}\left(\left|\eta (z)-\frac{1}{2}\right|\right)$. We measure the performance of a classifier h(z) under P relative to $h^{*}(z)$ using the excess risk, defined as:

$$L_{P}\left(h,h^{*}\right)=E_{P}\left[\ell (h)-\ell \left(h^{*}\right)\right],$$

where ℓ(h)=I{yh(z)≤0} is the 0–1 loss and I{⋅} denotes the indicator function. Similarly, for the perturbed distribution $P^{\left(\varepsilon \right)}$ with conditional probability function $\eta^{\left(\varepsilon \right)}(z)$, the corresponding Bayes classifier is $h^{*(\varepsilon )}(z)=\text{sign}\left\{\eta^{\left(\varepsilon \right)}(z)\geq 1/2\right\}$. The excess risk of a classifier h(z) under $P^{\left(\varepsilon \right)}$ is defined as:

$$L_{P^{\left(\varepsilon \right)}}\left(h,h^{*(\varepsilon )}\right)=E_{P^{\left(\varepsilon \right)}}\left[\ell (h)-\ell \left(h^{*(\varepsilon )}\right)\right].$$

Let $f^{\left(b\right)}(z)$ be a weak classifier trained from the noised data set ${\left\{\left(z_{i}^{\left(\varepsilon_{z}\right)},y_{i}^{\left(\varepsilon_{y}\right)}\right)\right\}}_{i=1}^{n_{0}}$. The next theorem bounds the excess risk of $f^{\left(b\right)}(z)$ under the unperturbed distribution P.

**Theorem 4** *Let* ℛ *be a fixed representation function from* 𝒳 *to* 𝒵. *Then for any weak classifier* $f^{\left(b\right)}$ *trained with* $n_{0}$ *samples from* $P^{\left(\varepsilon \right)}$,

$$L_{P}\left(f^{\left(b\right)},h^{*}\right)\leq L_{P^{\left(\varepsilon \right)}}\left(f^{\left(b\right)},h^{*(\varepsilon )}\right)+\frac{3}{4}d_{\text{TV}}\left(P_{Z},P_{Z}^{\left(\varepsilon \right)}\right)+E_{P_{Z}}\left[\left|\eta (z)-\eta^{\left(\varepsilon \right)}(z)\right|\right]+E_{P_{Z}}\left[\left|\eta (z)-\frac{1}{2}\right|-\left|\eta^{\left(\varepsilon \right)}(z)-\frac{1}{2}\right|\right],$$

*where*

$$d_{\text{TV}}\left(P_{Z},P_{Z}^{\left(\varepsilon \right)}\right)=2\;\underset{B\subseteq 𝒵}{\text{sup}}\;\left|P_{Z}(B)-P_{Z}^{\left(\varepsilon \right)}(B)\right|$$

*is the total variation distance between* $P_{Z}$ *and* $P_{Z}^{\left(\varepsilon \right)}$.

In Theorem 4, the term $L_{P^{\left(\varepsilon \right)}}\left(f^{\left(b\right)},h^{*(\varepsilon )}\right)$ captures the excess risk of $f^{\left(b\right)}$ under the privatized distribution $P^{\left(\varepsilon \right)}$. Since $f^{\left(b\right)}$ is trained on data from $P^{\left(\varepsilon \right)}$, this term reflects the inherent difficulty of the learning task under $P^{\left(\varepsilon \right)}$ and depends on the representation function ℛ, the choice of classification algorithm, and the training sample size $n_{0}$; see related theoretical studies in Blanchard et al. (2008); Sang et al. (2022); Ko et al. (2023). The following three terms quantify discrepancies between the original and privatized distributions and do not depend on the training sample size $n_{0}$. The total variation distance term $d_{\text{TV}}\left(P_{Z},P_{Z}^{\left(\varepsilon \right)}\right)$ measures the discrepancy between the marginal distributions of Z and $Z^{\left(\varepsilon_{z}\right)}$. Similar excess risk bounds involving a total variation distance term can be found in Ben-David et al. (2006). To further illustrate the magnitude of this term, Appendix A.4 provides a numerical illustration showing how $d_{\text{TV}}\left(P_{Z},P_{Z}^{\left(\varepsilon \right)}\right)$ varies with the dimension d and the privacy level $\varepsilon_{z}$. The term $E_{P_{Z}}\left[\left|\eta (z)-\eta^{\left(\varepsilon \right)}(z)\right|\right]$ quantifies how much the conditional distributions drift from η to $\eta^{\left(\varepsilon \right)}$, which can be seen as the discrepancy between the conditional distributions of Y∣Z and $Y^{\left(\varepsilon_{y}\right)}|Z$. The last term highlights the effect of deviation around the decision boundary 1/2, quantifying the increased uncertainty from the original distribution to perturbed distribution, where a narrower distance from 1/2 means more uncertainty. A similar term appears in the excess risk bound derived by Qin et al. (2024).

Recall from Section 3.4 that model reversal flips a classifier’s sign if it has estimated accuracy less than 0.5 under the original distribution P. Let $n_{1}$ be the sample size used for performance evaluation and $r^{\left(b\right)}$ be the true classification accuracy of $f^{\left(b\right)}$ under the distribution P. The following theorem shows how model reversal reduces the excess risk with high probability.

**Theorem 5** *Let* ℛ *be a fixed representation function from* 𝒳 *to* 𝒵. Suppose $f^{*(b)}$ *is obtained by selecting between* $\left\{f^{\left(b\right)},-f^{\left(b\right)}\right\}$ *via model reversal using* $n_{1}$ *evaluation samples. Then, with probability at least* $\Phi \left(\sqrt{n_{1}}\frac{\left|r^{\left(b\right)}-1/2\right|}{\sqrt{r^{\left(b\right)}\left(1-r^{\left(b\right)}\right)}}\right)$ *(where* Φ *is the cumulative distribution function of normal distribution),*

$$L_{P}\left(f^{*(b)},h^{*}\right)\leq L_{P^{\left(\varepsilon \right)}}\left(f^{\left(b\right)},h^{*(\varepsilon )}\right)+\frac{3}{4}d_{\text{TV}}\left(P_{Z},P_{Z}^{\left(\varepsilon \right)}\right)+2E_{P_{Z}}\left[\left|\eta (z)-\frac{1}{2}\right|-\left|\eta^{\left(\varepsilon \right)}(z)-\frac{1}{2}\right|\right].$$

Compared with the bound in Theorem 4, the term $E_{P_{Z}}\left[\left|\eta (z)-\eta^{\left(\varepsilon \right)}(z)\right|\right]\in [0,1]$ is replaced in Theorem 5 by $E_{P_{Z}}\left[\left|\eta (z)-\frac{1}{2}\right|-\left|\eta^{\left(\varepsilon \right)}(z)-\frac{1}{2}\right|\right]\in [0,1/2]$. When all perturbed datasets are non-negative (i.e., those with $g^{\left(\varepsilon \right)}(z)<1/2$), they share the same Bayes classifier as the original distribution, and $\left|\eta (z)-\eta^{\left(\varepsilon \right)}(z)\right|=\left|\eta (z)-\frac{1}{2}\right|-\left|\eta^{\left(\varepsilon \right)}(z)-\frac{1}{2}\right|$. In this case, the excess risk bounds in Theorems 4 and 5 coincide. In contrast, when some datasets are negative, model reversal leads to a tighter bound, demonstrating its added benefit in such scenarios. Moreover, since dataset utility is typically unknown in practice, Theorem 5 guarantees that with a sufficiently large evaluation sample size $n_{1}$, model reversal can effectively mitigate the influence of less-informative data and improve overall model performance.

Next, we analyze the final averaged classifier $f^{†}$ produced by combining B reversed weak classifiers ${\left\{f^{*(b)}\right\}}_{b=1}^{B}$ with weights determined by Equation (10). The following theorem provides a bound for its excess risk under P as B→∞.

**Theorem 6** *Let* ℛ *be a fixed representation function from* 𝒳 *to* 𝒵. For any (z,y)~P, *let* $F_{z}^{\left(\varepsilon \right)}$ *denote the distribution of classification accuracy* ${\tilde{r}}_{z}^{*(b)}=P\left\{\text{sign}\left[f^{*(b)}(z)\right]=y\right\}$ *under the random sampling of training and evaluation sets. Assume this distribution has support* $\left[r_{z,0},r_{z,1}\right]$ *with non-degenerate mass near* $r_{z,1}$. *Let* $f^{†}$ *be the model-averaged classifier defined in* Equation (11), *and let* $B_{0}$ *be the number of classifiers exceeding the cutoff* $r_{0}$. *If* $r_{0}$ *is chosen such that* $B_{0}/B\to 0$ *as* B→∞, *then*

$$\underset{B\to \infty }{\text{lim}\;\text{sup}}L_{P}\left(f^{†},h^{*}\right)\leq L_{P^{\left(\varepsilon \right)}}\left(f^{†},h^{*(\varepsilon )}\right)+\frac{3}{4}d_{\text{TV}}\left(P_{Z},P_{Z}^{\left(\varepsilon \right)}\right)+2E_{P_{Z}}\left[\left|\eta (z)-\frac{1}{2}\right|+\frac{1}{2}-r_{z,1}\right],\;\text{in}\;\text{probability}.$$

This result shows that the excess risk of the model-averaged classifier is asymptotically bounded in probability bounded by a quantity that depends on the best-performing classifiers, i.e., those near the upper end $r_{z,1}$ of the accuracy distribution. As the number of classifiers B increases, the weight assigned to lower-performing models vanishes under the thresholding scheme in Equation (10), leading the ensemble to concentrate on top-performing classifiers. This effectively implements a form of regularized extreme value selection. The convergence rate of the bound depends on the amount of probability mass that $F_{z}^{\left(\varepsilon \right)}$ places near $r_{z,1}$; see Leadbetter et al. (2012) for related results from extreme value theory. The third term in the bound reflects the remaining gap between the best achievable accuracy $r_{z,1}$ and the Bayes-optimal level $\left|\eta (z)-\frac{1}{2}\right|+\frac{1}{2}$. Compared to Theorem 5, this bound is tighter when high-quality classifiers exist and can be identified reliably through evaluation. These insights are consistent with our empirical findings in Section 6, where model averaging improves performance, especially in high-noise regimes.

While the above results focus on a single-server setting, they naturally extend to multi-server scenarios introduced in Section 3.6. In a heterogeneous multi-server environment, each server can treat other servers’ classifiers as additional weak classifiers and apply model reversal and model averaging locally. The same type of bounds hold, subject to analogous conditions for data partition and accuracy estimation.

Deciding how to split data between the training and evaluation sets, as well as how many weak classifiers to train, is crucial. The theoretical results above provide guidance: a sufficiently large training sample size $n_{0}$ is necessary to ensure that each weak classifier is adequately learned, achieving lower excess risk $L_{P^{\left(\varepsilon \right)}}\left(f^{\left(b\right)},h^{*(\varepsilon )}\right)$; a larger evaluation sample size $n_{1}$ improves the precision of utility estimates, thereby strengthening model reversal; and a larger number of weak classifiers B leads to a more stable aggregated classifier, enhancing the effect of model averaging. Meanwhile, our empirical findings (see Appendix A.2) suggest that in high-noise regimes, it may be more beneficial to allocate more samples to evaluation rather than training.

## 5. Functional Data Classification under LDP

Advances in sensing technologies have made it increasingly feasible to collect data densely sampled over temporal or spatial domains, giving rise to what is commonly referred to as functional data (Ramsay and Silverman, 2005). Examples include physiological signals such as heart rate and electrocardiogram readings from wearable devices, GPS traces from smartphones that capture mobility patterns, and sensor data from activity trackers monitoring daily routines or sleep cycles. These data streams, while informative, can also expose sensitive information related to an individual’s health, behavior, and lifestyle. This makes privacy preservation particularly critical in biomedical and health-monitoring applications, where temporal or spatial patterns may reveal personal characteristics or preferences (Stisen et al., 2015). Despite its importance, privacy-preserving learning with functional data remains largely unexplored. As a concrete demonstration of our approach, this section presents the full pipeline for constructing a classifier for functional data under LDP. To the best of our knowledge, this is the first framework for functional classification under LDP.

Suppose there is a single server with access to N clients. Each client holds a square-integrable functional covariate x(t), defined on a compact domain ℐ=[0,1], and a corresponding binary label y∈{-1,1}. Our goal is to learn a functional linear classifier

$$f\left(x\right)=\alpha +\int_{0}^{1}x\left(t\right)\beta \left(t\right)\;\text{dt},$$

such that we predict $\overset{ˆ}{y}(x)=\text{sign}\{f(x)\}$. In accordance with Yang et al. (2020), an LDP algorithm typically proceeds in four stages: encoding, perturbation, aggregation, and estimation. Below, we describe how each stage applies to functional data.

### 5.1 Encoding and Perturbation

For each client with a functional covariate x(t) and label y in the training set, the data reported to the server is transformed through the following steps.

#### Dimensionality Reduction.

Functional data are often observed on a fine grid of time points (or locations), leading to high-dimensional vectors. To reduce dimensionality in a privacy-preserving way, we project x(t) onto a finite set of basis functions ${\left\{\phi_{k}(t)\right\}}_{k=1}^{d}$, which is corresponding to the representation process in Section 3.1. Concretely,

(12) $$x\left(t\right)=\sum_{k=1}^{d}z_{k}\phi_{k}\left(t\right)+\xi \left(t\right),$$

where $z={\left(z_{1},\ldots ,z_{d}\right)}^{⊤}\in R^{d}$ are the basis coefficients and ξ(t) is a residual term excluded from the model. The choice of basis functions $\Phi =\left(\phi_{1},\ldots ,\phi_{d}\right)$ (e.g., B-splines, Fourier bases) and the dimension d are specified by the server, where d controls the representation fidelity and also affects the magnitude of the subsequent privatization for a fixed privacy budget. This projection serves two key purposes:

1. Functional data fitting. In practice, x(t) is discretely observed at T time points, $x={\left(x\left(t_{1}\right),\ldots ,x\left(t_{T}\right)\right)}^{⊤}$. For smooth families like B-splines of order m, fitting accuracy improves at the optimal rate of $T^{-2m/(2m+1)}$ as the number of knots grows like $T^{1/(2m+1)}$ (Eubank, 1999), which indicates a relatively slow rate of increase for d.
2. Privacy enhancement. Truncating at d dimensions mitigates overfitting to person-specific variations, thereby enhancing privacy. It also significantly reduces communication overhead compared to transmitting the full functional data.

When the chosen basis cannot adequately represent the underlying signal, or when the classification rule is too complex to be well approximated by a basis expansion, the separate perturbation mechanism in Equation (1) may lead to substantial information loss. This mechanism can be viewed as inducing a form of measurement error in both covariates and labels, and classification performance may deteriorate further in such settings (Li and Ma, 2024). More generally, Equation (1) can be replaced by more sophisticated LDP mechanisms, together with the corresponding algorithms for constructing weak classifiers (Berrett and Butucea, 2019; Ma and Yang, 2024), while our proposed evaluation, model reversal, and model averaging procedures remain applicable.

#### Rescaling.

To bound sensitivity for LDP, we rescale each component of z into a fixed range, typically [−1, 1]. We consider two simple examples:

$$\left(\text{Tanh}\;\text{Transformation}\right)\;z_{k}^{*}=\text{tanh}\left(z_{k}\right),$$

$$\left(\text{Max-Abs}\;\text{Normalization}\right)\;z_{k}^{*}=\frac{z_{k}}{{\text{max}}_{k^{\prime }}\;\left|z_{k^{\prime }}\right|}.$$

Here, $z^{*}={\left(z_{1}^{*},\ldots ,z_{d}^{*}\right)}^{⊤}\in [-1,1{]}^{d}$. The tanh transformation is applied componentwise and introduces a nonlinear squashing effect, whereas the Max-Abs normalization is a linear rescaling that preserves coefficient signs and the relative magnitudes across coordinates. Other bounded transformations can also be used to map the feature vector into a fixed bounded range and thereby control sensitivity under the chosen LDP mechanism. In Appendix A.1, we compare these two rescaling choices and observe similar classification accuracy in our experimental setting. Therefore, for concreteness, the experiments in Section 6 report results based on the tanh transformation.

#### Perturbation.

After rescaling, each client adds noise according to an LDP mechanism. Using a Laplace mechanism (Dwork et al., 2006), they report

(13) $$z^{\left(\varepsilon_{z}\right)}=z^{*}+\delta^{\left(\varepsilon_{z}\right)},$$

where $\delta^{\left(\varepsilon_{z}\right)}\in R^{d}$ has independent and identically distributed (i.i.d.) Laplace entries with scale $\lambda =d\Delta /\varepsilon_{z}$ and Δ=2 (since each $z_{k}^{*}\in [-1,1]$). Hence each coordinate’s privacy budget is effectively $\varepsilon_{z}/d$. To protect the binary label y, we apply the randomized response mechanism (Warner, 1965):

(14) $$P\left(y^{\left(\varepsilon_{y}\right)}=y\right)=\frac{e^{\varepsilon_{y}}}{1+e^{\varepsilon_{y}}},$$

where $\varepsilon_{y}=\varepsilon -\varepsilon_{z}$. In the experiment, we choose $\varepsilon_{y}=\varepsilon /(d+1)=\varepsilon_{z}/d$. Theorem 7 shows that the entire encoding and perturbation procedure satisfies ε-LDP.

**Theorem 7** *Let* ${ℳ}_{f}$ *be the privacy mechanism taking* (x(t),y) *as input and returning* $\left(z^{\left(\varepsilon_{z}\right)},y^{\left(\varepsilon_{y}\right)}\right)$ *via* Equations (13)–(14). *Then* ${ℳ}_{f}$ *satisfies* ε-*LDP*.

It is worth noting that we employ the Laplace mechanism here due to its simplicity and ease of interpretation, which facilitates our subsequent demonstrations of the proposed methods. As shown in Duchi et al. (2018), adding Laplacian noise to (appropriately truncated) observations is an optimal privacy mechanism in one-dimensional mean estimation under LDP. However, this optimality does not directly extend to higher-dimensional settings. For additional insights on such mechanisms, we refer readers to Nguyên et al. (2016); Duchi et al. (2018); Wang et al. (2019a); Xiao et al. (2023).

### 5.2 Classifier Construction with MRMA

Once the server collects ${\left\{\left(z_{i}^{\left(\varepsilon_{z}\right)},y_{i}^{\left(\varepsilon_{y}\right)}\right)\right\}}_{i=1}^{N_{0}}$ from $N_{0}$ training clients, it can train multiple weak classifiers as introduced in Section 3.3. Two general methods are outlined below.

#### Method I.

It’s straightforward to build a vector classifier by treating $z_{i}^{\left(\varepsilon_{z}\right)}\in R^{d}$ as the input to a standard classification model (e.g., logistic regression, SVM). Denote the estimated intercept and coefficients by $\overset{ˆ}{\alpha }$ and $\overset{ˆ}{b}={\left({\overset{ˆ}{b}}_{1},\ldots ,{\overset{ˆ}{b}}_{d}\right)}^{⊤}\in R^{d}$, respectively. We then recover the slope function via $\hat{\beta }(t)=\sum_{k=1}^{d}{\overset{ˆ}{b}}_{k}\phi_{k}(t)$, and define the corresponding functional classifier as

$$\overset{ˆ}{f}\left(x\right)=\overset{ˆ}{\alpha }+\int_{0}^{1}x\left(t\right)\overset{ˆ}{\beta }\left(t\right)\;\text{dt}.$$

#### Method II.

Before building the classifier, we first reconstruct the function using the perturbed basis scores, i.e., treating $x_{i}^{\left(\varepsilon_{z}\right)}(t)=\sum_{k=1}^{d}z_{i,k}^{\left(\varepsilon_{z}\right)}\phi_{k}(t)$ as the perturbed functional covariate, where $z_{i,k}^{\left(\varepsilon_{z}\right)}$ represents the kth element of $z_{i}^{\left(\varepsilon_{z}\right)}$. Then, the classifier can be obtained using the functional conjugate gradient algorithm (CG, Kraus and Stefanucci, 2019), functional distance weighted discrimination (DWD, Sang et al., 2022), or any other established functional classification method.

With these two ways of constructing classifiers, in the Appendix A.1, we illustrate the effects of dimensionality reduction, rescaling, and perturbation on the misclassification rate of the classifiers. Experiment results indicate that the projection of functional data and rescaling of coefficient vectors have a small impact on the classifier’s performance. And the performance of two types of transformations is similar in our context. Moreover, as the privacy budget ε→0, the misclassification rates of the weak classifiers based on the perturbed data tend to 50%.

#### MRMA.

Based on the noised binary evaluations collected from the additional $N_{1}$ evaluation clients in Section 3.3, we apply the MRMA procedure introduced in Section 3.4. Specifically, for a weak classifier $f^{\left(b\right)}$, if its estimated accuracy ${\tilde{r}}^{\left(b\right)}<50\%$, we invert its parameters:

$$\left({\overset{ˆ}{\alpha }}^{\left(b\right)},{\hat{\beta }}^{\left(b\right)}(t)\right)⟼\left(-{\overset{ˆ}{\alpha }}^{\left(b\right)},-{\hat{\beta }}^{\left(b\right)}\left(t\right)\right).$$

Let ${\left\{f^{*(b)}\right\}}_{b=1}^{B}$ be the reversed classifiers, and denote their weights by ${\left\{w_{b}\right\}}_{b=1}^{B}$ as in Equation (10). Our final averaged functional classifier becomes

$$f^{†}\left(x\right)={\overset{ˆ}{\alpha }}^{†}+\int_{0}^{1}x\left(t\right){\hat{\beta }}^{†}\left(t\right)\;\text{dt},$$

where ${\overset{ˆ}{\alpha }}^{†}=\sum_{b=1}^{B}w_{b}{\overset{ˆ}{\alpha }}^{*(b)},{\hat{\beta }}^{†}(t)=\sum_{b=1}^{B}w_{b}{\hat{\beta }}^{*(b)}(t)$.

This example demonstrates how our LDP framework generalizes naturally to infinite-dimensional data via basis truncation, preserving both privacy and utility. It highlights the method’s flexibility in accommodating complex data structures.

## 6. Experiments

In this section, we present experiments that primarily demonstrate the improvements in classification accuracy achieved by applying our proposed techniques, compared to baselines that do not use them. These results validate the effectiveness of our approach. Differences in performance across classifier types are due to their intrinsic properties and are not the focus of this study.

In each experimental trial, the functional covariate X(⋅) is generated by $X(t)=\sum_{j=1}^{50}\xi_{j}\zeta_{j}\phi_{j}(t)$ for t∈[0,1], where $\xi_{j}$’s are independently drawn from $U(-\sqrt{3},\sqrt{3})$, $\zeta_{j}=(-1{)}^{j+1}j^{-1},j=1,\ldots ,50,\phi_{1}(t)=1$ and $\phi_{j}(t)=\sqrt{2}\;\text{cos}((j-1)\pi t)$ with j≥2. The binary response variable Y, taking values 1 or −1, is generated using the following logistic model:

$$f\left(X\right)=\alpha_{0}+\int_{0}^{1}X\left(t\right)\beta \left(t\right)\text{dt},\;\text{Pr}\left(Y=1\right)=\frac{\text{exp}\left\{f\left(X\right)\right\}}{1+\text{exp}\left\{f\left(X\right)\right\}},$$

where $\alpha_{0}=0.1,\beta (t)$ is the slope function, and f(X) is referred to as the classification function. Results in Appendix A.1 show that the performance with d=4,5,6 cubic B-Spline is comparable, and the performance based on Tanh or Max-Abs transformation is close. Thus we present results with d=4 and employ the tanh transformation.

### 6.1 Single-Server Classification with MRMA

In this section, we demonstrate the improvements in classification accuracy brought about by model reversal and model averaging. We generate data for a server using the slope function $\beta (t)=\sum_{j=1}^{50}4(-1{)}^{j+1}j^{-2}\phi_{j}(t)$. Assuming the server has a total of N=3000 clients, we allocate $N_{0}=500$ for training and the remaining $N_{1}=2500$ for evaluation. To construct classifiers, we sequentially draw $n_{0}=50$ samples from the training data set without replacement, repeating this procedure B=50 times. At the same time, we partition the evaluation set into B=50 subsets of equal size, enabling us to evaluate the classification accuracy of each classifier using $n_{1}=50$ distinct samples. To assess the performance of the classifiers, we randomly generate a testing data set comprising 500 samples during each trial, repeating this procedure 500 times.

**Figure 2:**
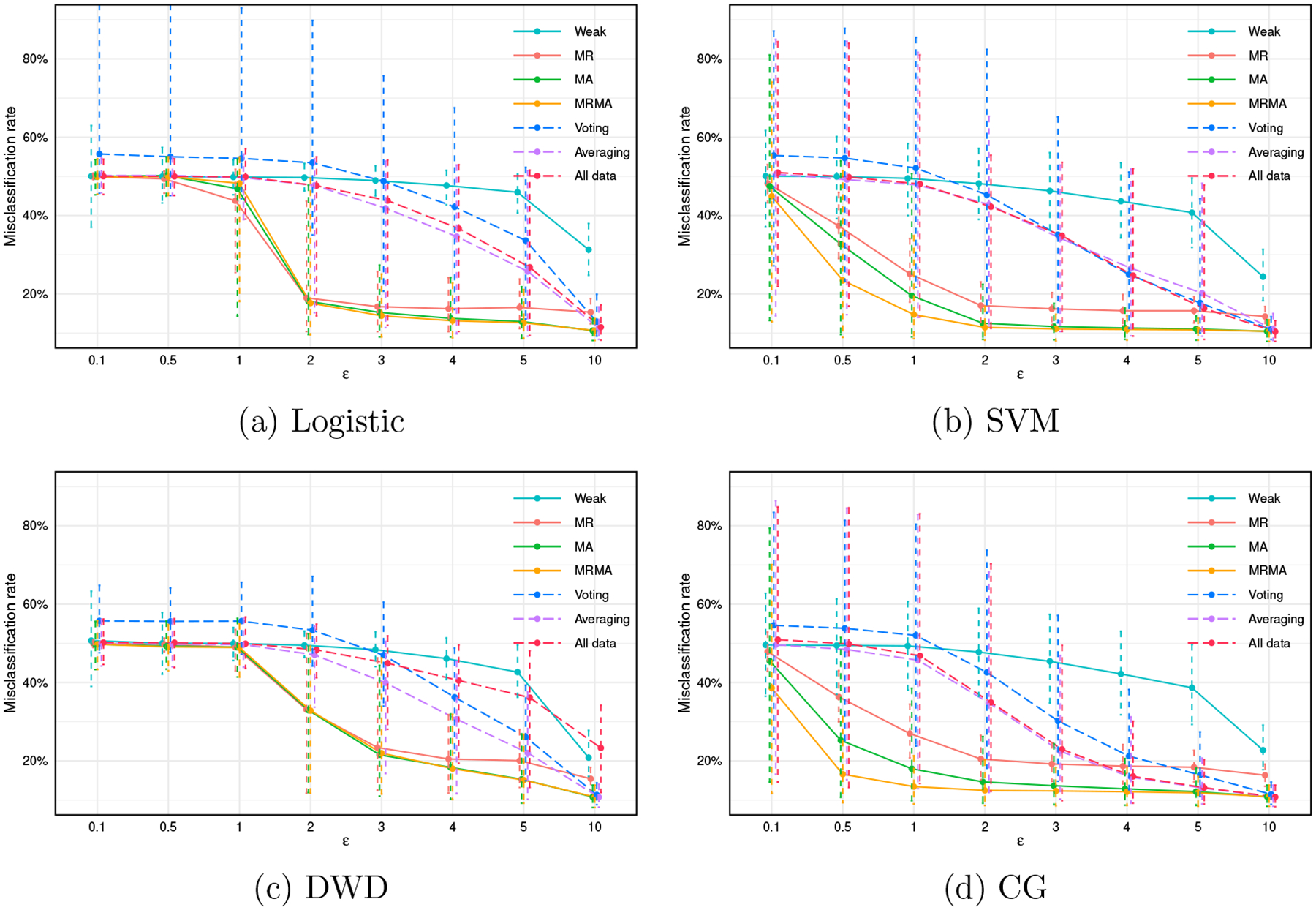
The misclassification rates of classifiers with a single server under ε-LDP.

Figure 2 showcases the misclassification rates along with error bars, for various classifiers across different ε levels. In this figure, “Weak” denotes the average misclassification rate of B=50 weak classifiers obtained through sampling. “MR” represents the average misclassification rate of B weak classifiers after model reversal under LDP. “MA” signifies the results when using model averaging on weak classifiers with cutoff value $r_{0}=0.8$, while “MRMA” illustrates the results of applying both model reversal and model averaging. To compare with classic ensemble methods, we train B weak classifiers under LDP. Each classifier is trained with N/B=60 instances from the total sample. We then obtain the results through majority voting and equal-weight averaging, denoted as “Voting” and “Averaging”, respectively. And “All data” denotes the classifier trained with N clients directly.

**Figure A.5:**
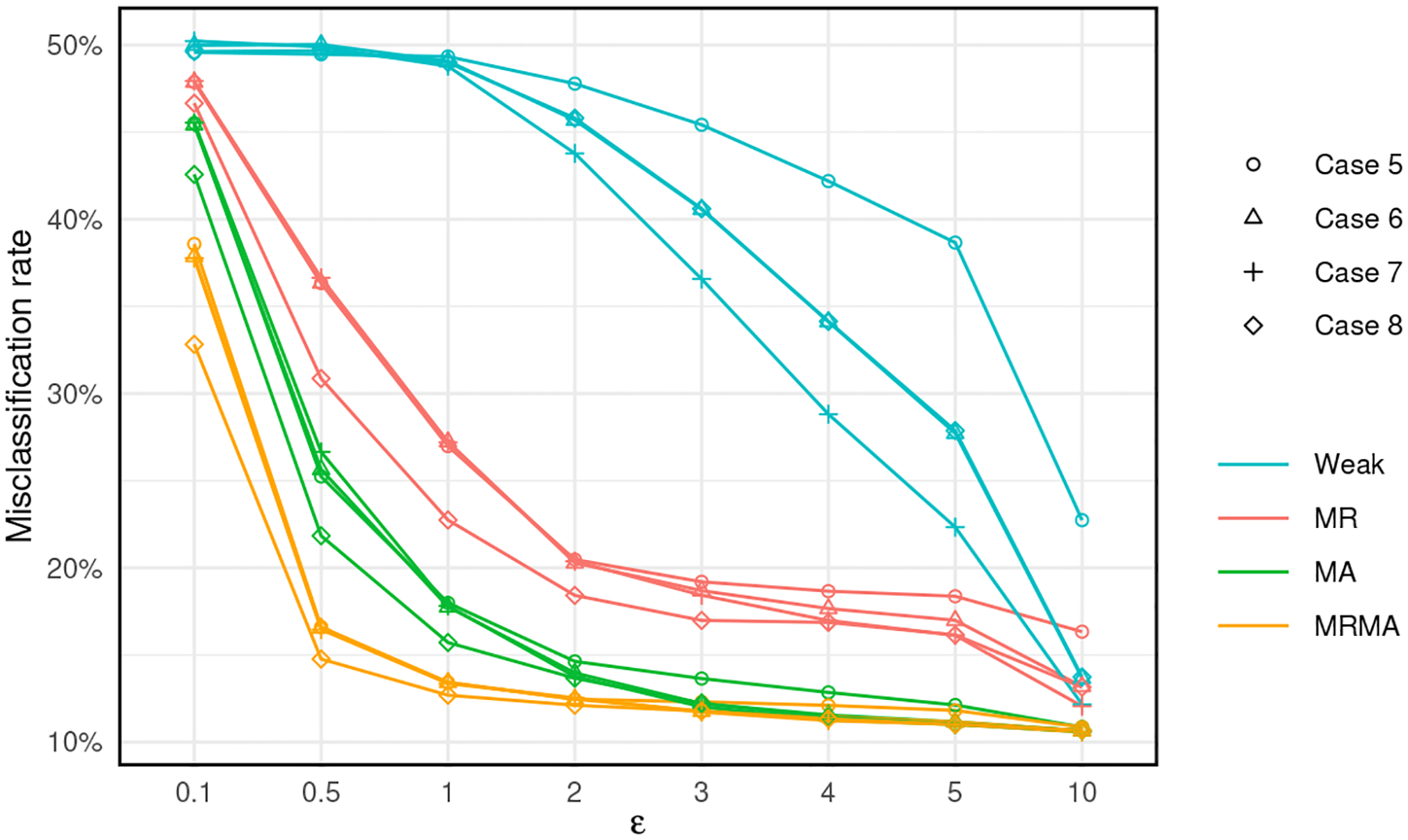
The misclassification rates of classifier CG with a single server under ε-LDP.

The classifier “All data”, even if it is trained with 3000 clients, shows almost no improvement over the classifier “Weak” when ε is small (indicating substantial noise interference). And classifiers “Voting” and “Averaging” also perform similarly. However, our proposed techniques, both model reversal and model averaging, significantly improve the performance of all types of weak classifiers. And MRMA further enhances the performance of SVM and CG classifiers substantially. Figure A.5 in Appendix A.3 demonstrates that even when allocating more clients for training weak classifiers, MR and MA can still significantly enhance the classifiers’ performance. From the client’s perspective, this means that to achieve a target classification accuracy, our method allows for a higher level of privacy protection, that is, a smaller value of ε can be used without sacrificing performance. For further discussions regarding distinctions among different types of classifiers, please refer to Appendix A.1.

### 6.2 Multi-Server Classification with MRMA under Heterogeneity

In this section, we showcase the improvements in classification accuracy achieved through multi-server learning for individual servers. We consider a total of K=25 servers, divided into three groups, each characterized by a distinct slope function to introduce heterogeneity. Specifically,

- Group 1: for $k=1,\ldots ,10,\beta_{k}(t)=\sum_{j=1}^{50}\gamma_{j}(-1{)}^{j+1}j^{-2}\phi_{j}(t)$ with $\gamma_{j}\overset{\text{i.i.d.}}{\sim }U(-8,-2)$.
- Group 2: for $k=11,\ldots ,15,\beta_{k}(t)\sim \text{GP}(0,K(s,t))$ with K(s,t)=exp(-15|s-t|).
- Group 3: for $k=16,\ldots ,25,\beta_{k}(t)=\sum_{j=1}^{50}\gamma_{j}(-1{)}^{j+1}j^{-2}\phi_{j}(t)$ with $\gamma_{j}\overset{\text{i.i.d.}}{\sim }U(2,8)$.

Here, GP(0,K(s,t)) represents a Gaussian process with zero mean and kernel K(s,t). It is essential to highlight that the slope functions of servers within the same group are not identical. Moreover, the directions of the slope functions in groups 1 and 3 are opposite, serving as a test to assess the potential negative transfer impact during multi-server learning. In group 2, the slope function is randomly generated, resulting in an approximate 50% misclassification rate for classifiers built on servers in this group. This design is purposefully implemented to gauge its potential disruption to the multi-server learning process.

**Figure 3:**
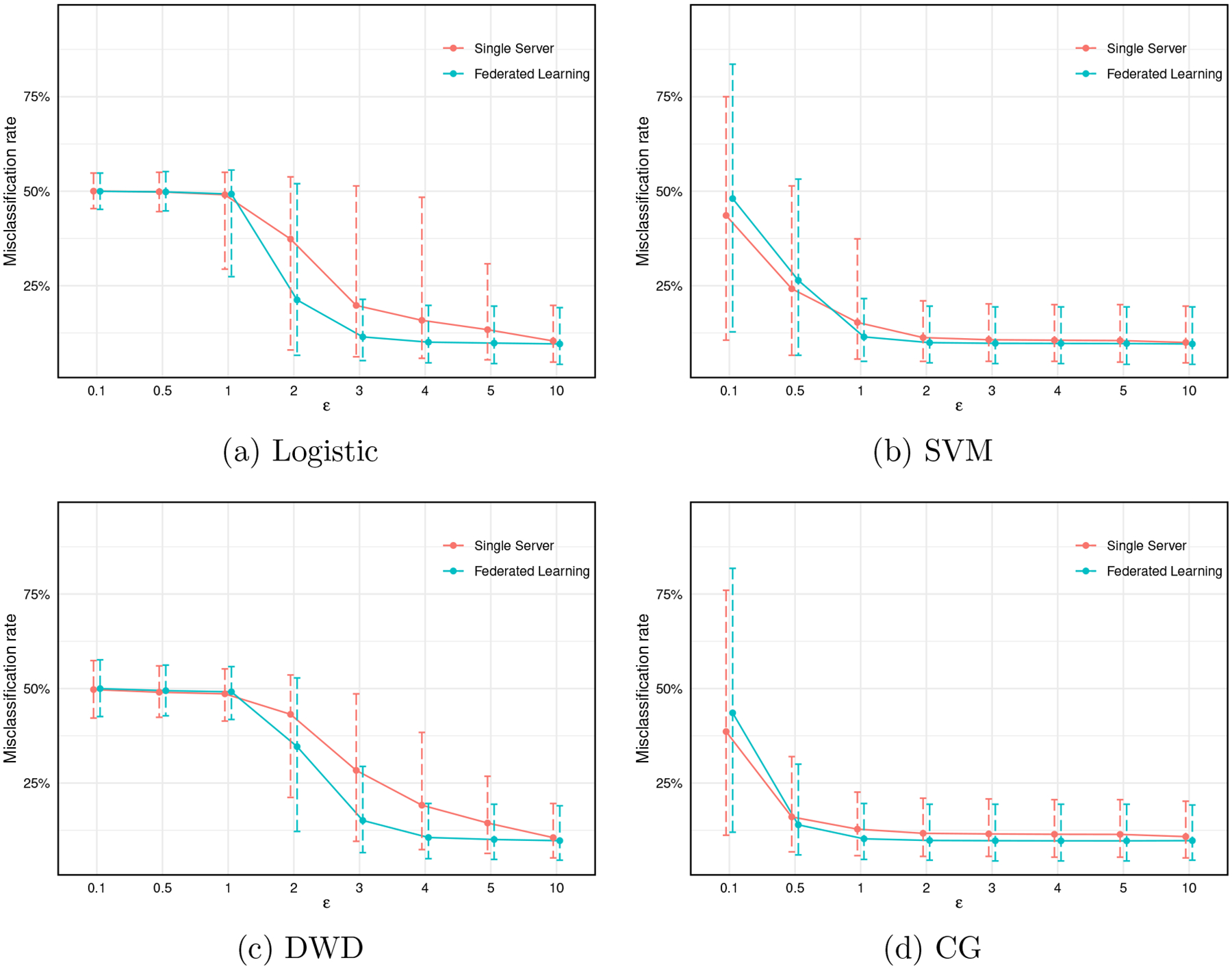
The misclassification rates of classifiers with multi-server under ε-LDP.

For each server, we generate N=3000 clients, with an additional 500 clients designated for testing. Each server employs algorithms with the parameters $N_{0}=500,N_{1}=2500,n_{0}=50$, and B=50. Initially, each server independently obtains its own classifier using MRMA, with parameters set to $\varepsilon_{v}=\varepsilon$ and $r_{0}=0.8$. Subsequently, all servers proceed to perform multi-server learning. The parameters for this phase are set as $\varepsilon_{v}=\varepsilon_{v}^{*}=\varepsilon /2$, with $r_{0}^{*}=0.8$ to mitigate potential negative transfer effects. As expected, servers in group 2 exhibit misclassification rates around 50%. The average misclassification rates for classifiers of servers in groups 1 and 3 under these two scenarios are illustrated in Figure 3. The results show that multi-server learning significantly improves the performance of both logistic and DWD classifiers, even with server heterogeneity. While SVM and CG classifiers already perform well within a single server setting, multi-server learning shows comparable or slightly better performance.

## 7. Real Application

To demonstrate the practical utility of our proposed classification methods under LDP, we evaluate their performance on both vector-valued and functional datasets, each involving sensitive information that necessitates privacy protection. Section 7.1 presents results from two real-world datasets with vector predictors related to diabetes and employee attrition, while Section 7.2 focuses on applications involving functional predictors derived from physical activity and speech recordings.

### 7.1 Vector-valued Predictors

We first evaluate our methods on two publicly available datasets: a healthcare dataset related to diabetes risk and an employee attrition dataset. Both datasets contain sensitive information, making them suitable candidates for privacy-preserving classification. We compare our methods with the histogram-based classifier under LDP proposed by Berrett and Butucea (2019), referred to as “Histogram”. We fix the number of splits to $h^{-1}=4$ along each axis, since tuning h under LDP would require an additional privacy-preserving validation step and further complicate the procedure.

The Diabetes dataset, published by Pore (2023), comprises 2768 samples and includes eight health-related attributes used to predict whether an individual has diabetes. We randomly split the dataset into 2214 training samples (approximately 80%) and 554 testing samples. To assess classifier performance under LDP, we repeat this process 500 times. In each repetition, we draw $N_{0}=414$ samples from the training samples to serve as the training set, while the remaining $N_{1}=1800$ samples are used for evaluation. Specifically, we randomly sample $n_{0}=60$ observations (without replacement) from the training set to construct a classifier, repeating this procedure B=30 times to generate multiple weak classifiers. Then we partition the evaluation set into B=30 subsets of equal size, enabling us to evaluate the classification accuracy of each classifier using $n_{1}=60$ distinct samples.

The Employee dataset, introduced by Elmetwally (2023), contains employment-related records for 4653 individuals, each described by eight variables, including education, job history, and workplace factors. The task is to predict whether an employee leaves the company. We split the dataset into 3722 training samples and 931 testing samples, again repeating the process 500 times. Within each repetition, we use $N_{0}=722$ for training and $N_{1}=3000$ for evaluation. Classifier construction and evaluation follow the same procedure as described for the Diabetes dataset, using $n_{0}=n_{1}=100$ and B=30.

Tables 1 and 2 report the average misclassification rates and standard deviations across 500 repetitions under various privacy levels (ε) for both datasets. We set $r_{0}=0.7$ to ensure the existence of weak classifiers satisfying this criterion. Our MRMA-based classifier consistently achieves superior accuracy, particularly when ε is small, corresponding to stronger privacy guarantees. As ε increases, indicating weaker privacy constraints, the performance differences among our proposed methods, the ensemble strategies (“Voting” and “Averaging”), and the “All data” method become negligible, with all achieving similar accuracy and significantly outperforming the histogram-based approach. Notably, even when ε=1000, the histogram method remains less effective, likely due to the substantial Laplace noise introduced, whose scale is $2^{d+1}/\varepsilon$, where d=8 is the number of predictors. This level of noise, although reduced for large ε, still introduces enough distortion to degrade classification performance.

### 7.2 Functional Predictors

We evaluate our LDP methods on two real-world classification tasks involving functional predictors. The first task uses wearable-sensor-based physical activity data to predict cardiovascular health status, while the second revisits the phoneme classification problem using frequency-domain speech features.

The first task draws on data from the National Health and Nutrition Examination Survey 2013–2014 cycle. We consider adult female participants aged 20 years or older, for whom both laboratory-measured high-density lipoprotein (HDL) cholesterol and minute-level physical activity recordings are available. HDL is known to be protective against cardiovascular disease, and its levels are influenced by physical activity. According to clinical guidelines, HDL is considered healthy for women when its concentration is at least 50 mg/dL (Turk et al., 2009). Using this threshold, we define a binary classification task to distinguish participants with healthy versus unhealthy HDL levels. As the functional predictor, we use hour-level Monitor-Independent Movement Summary (MIMS) values averaged over seven days and projected onto a set of 6 cubic B-spline basis functions. The resulting dataset includes 2410 samples, with 1558 labeled as healthy and 852 as unhealthy. To evaluate classifier performance under LDP, we adopt a randomized split: 410 samples are held out as a test set, and the remaining 2000 are divided into a training set of size $N_{0}=500$ and an evaluation set of size $N_{1}=1500$. For each repetition, we subsample $n_{0}=n_{1}=50$ observations to train and evaluate classifiers across B=30 randomized subsets. This entire process is repeated 500 times.

The second task uses the Phonemes dataset derived from the TIMIT Acoustic-Phonetic Continuous Speech Corpus (Garofolo, 1993). Speech frames in this dataset are extracted from the continuous speech of 50 male speakers, and log-periodograms are constructed from recordings sampled at 256 equispaced frequencies and projected onto a set of 4 Fourier basis functions. The classification task focuses on distinguishing between the phonemes “sh” and “iy”, represented by 1163 and 872 curves, respectively. The log-periodogram functions are approximated using Fourier basis expansion. As in the MIMS example, we adopt a randomized split of 535 test samples, a training set of $N_{0}=300$, and an evaluation set of $N_{1}=1200$, with $n_{0}=n_{1}=50$, and B=24, repeating the entire process 500 times.

**Figure 4:**
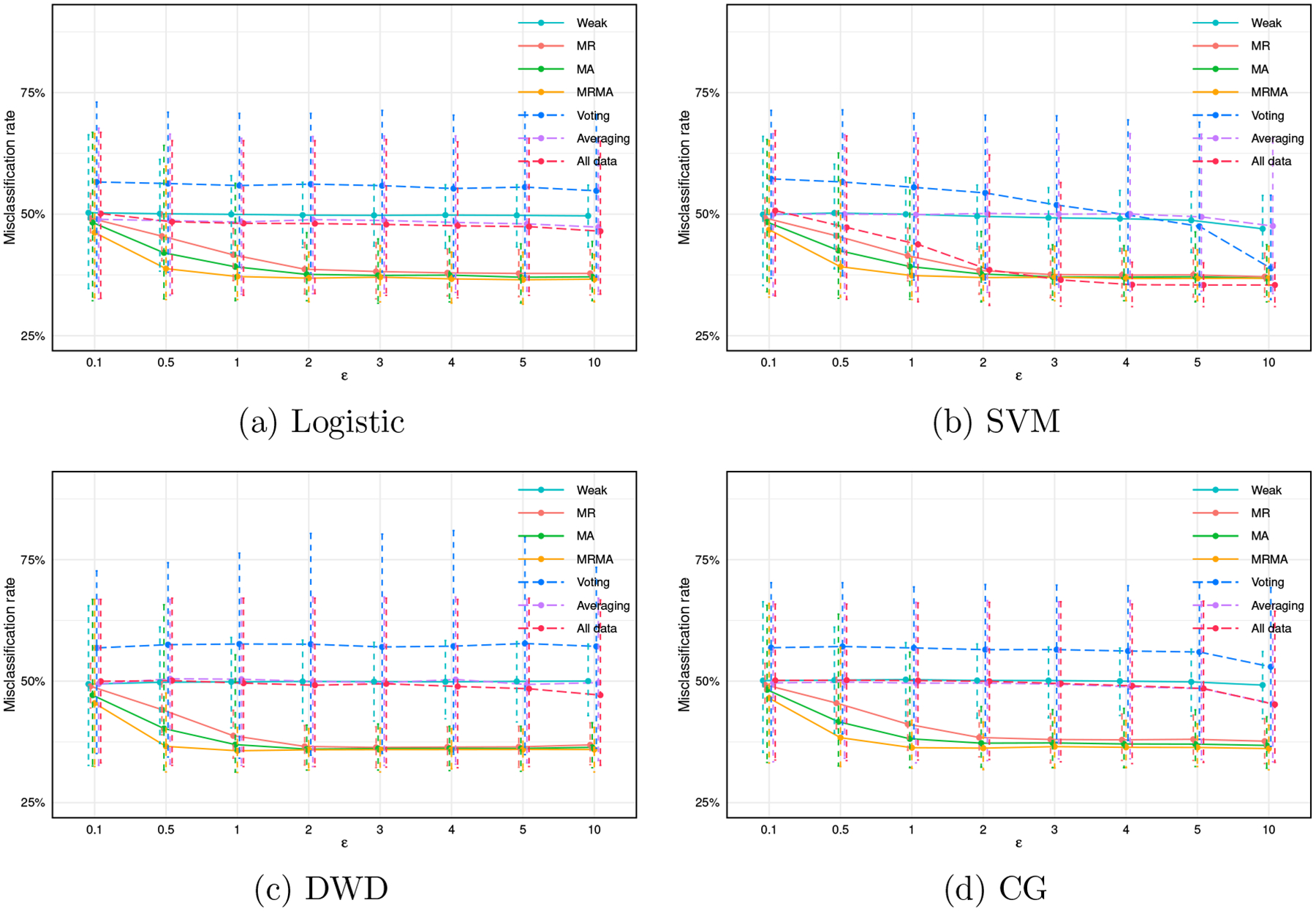
The misclassification rates of classifiers with a single server under ε-LDP on the physical activity dataset.

**Figure 5:**
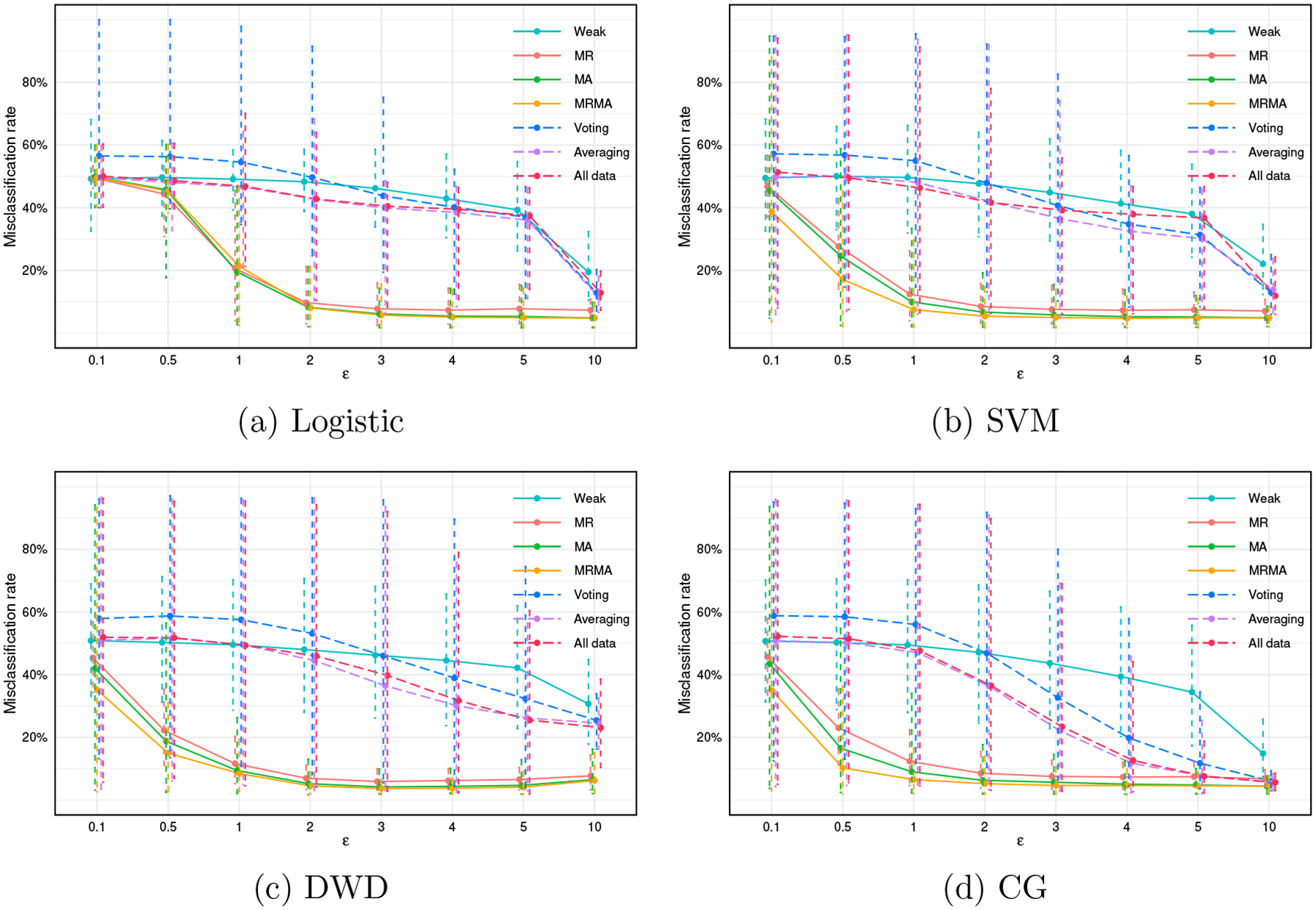
The misclassification rates of classifiers with a single server under ε-LDP on the Phonemes dataset.

**Figure A.7:**
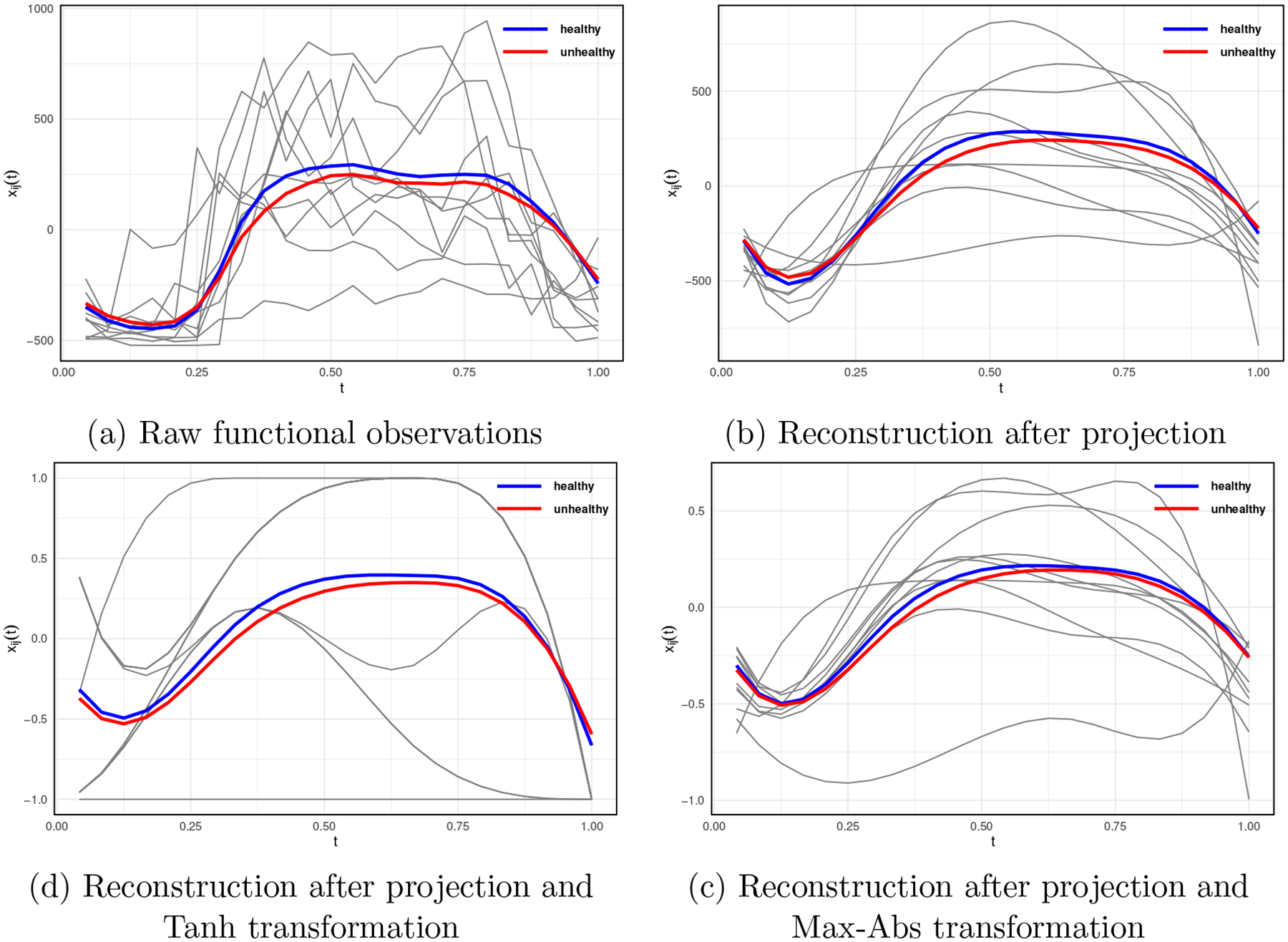
Visualization of the mean MIMS curves for the “healthy” (HDL ≥ 50) and “unhealthy” (HDL < 50) groups in the physical activity dataset, with 10 randomly selected individual curves overlaid in gray. Each subplot shows the curves under different stages of processing the raw functional data.

Visualizations of the functional predictors in these two applications are provided in Figures A.7–A. 8 in the Appendix. These figures display both the raw functional observations and the curves reconstructed after finite basis projection and transformation. Despite some smoothing and loss of fine-grained detail, the figures demonstrate that the key temporal patterns and population-level group differences are well preserved. Figures 4–5 report their misclassification rates. The parameter $r_{0}$ is set to 0.7 for the physical activity dataset and 0.9 for the Phonemes dataset. In both examples, classifiers based on model reversal and model averaging achieve substantial performance gains compared to other classifiers.

## 8. Conclusion

We proposed a novel framework for classification under LDP, addressing key challenges in data utility and model performance. By reinterpreting private learning as a transfer learning problem, we introduced a utility evaluation mechanism based on privatized binary feedback, enabling accurate assessment of classifier performance without accessing unperturbed data. Building on this, we developed two techniques: model reversal, which rescues underperforming classifiers by inverting decision boundaries, and model averaging, which combines multiple weak classifiers using utility-based weights. Together, these methods form a robust strategy, MRMA, that significantly enhances classification accuracy under LDP constraints. We provided theoretical excess risk bounds demonstrating the effectiveness of these techniques, and showed that the framework generalizes naturally to functional data settings through basis projection. Empirical evaluations on both simulated and real-world datasets confirmed substantial improvements over baseline approaches. Future directions include further theoretical analysis of specific classifiers under particular LDP mechanisms, as well as extending the framework to multi-class classification, regression, and other structured data modalities under LDP constraints.

## Figures and Tables

**Table 1:** Mean and standard deviation (in parentheses) of misclassification rates for various classification methods, computed over 500 random sample splits of the Diabetes dataset and evaluated under different values of ε for ε-LDP, with bold values indicating the best performance.

ε	Weak	MR	MA	MRMA	Voting	Averaging	All data	Histogram
0.1	49.55 (0.078)	48.22 (0.032)	46.22 (0.153)	**43.44** (0.144)	55.25 (0.221)	49.03 (0.157)	50.82 (0.158)	49.75 (0.057)
0.5	49.23 (0.066)	40.23 (0.031)	37.32 (0.095)	**34.49** (0.030)	53.93 (0.226)	45.61 (0.152)	46.33 (0.153)	49.75 (0.057)
1	48.48 (0.055)	34.90 (0.020)	34.59 (0.033)	**34.31** (0.018)	48.09 (0.197)	41.50 (0.132)	41.57 (0.132)	49.75 (0.057)
5	42.95 (0.047)	34.16 (0.017)	**34.31** (0.018)	**34.31** (0.018)	34.38 (0.019)	**34.31** (0.018)	**34.31** (0.018)	49.64 (0.057)
10	38.06 (0.030)	**34.01** (0.018)	34.29 (0.018)	34.30 (0.018)	34.31 (0.018)	34.31 (0.018)	34.31 (0.018)	49.58 (0.058)
1000	26.60 (0.010)	26.02 (0.013)	23.03 (0.018)	23.03 (0.018)	22.81 (0.016)	22.23 (0.016)	**22.05** (0.015)	39.80 (0.036)

**Table 2:** Mean and standard deviation (in parentheses) of misclassification rates for various classification methods, computed over 500 random sample splits of the Employee dataset and evaluated under different values of ε for ε-LDP, with bold values indicating the best performance.

ε	Weak	MR	MA	MRMA	Voting	Averaging	All data	Histogram
0.1	49.71 (0.082)	47.86 (0.031)	46.43 (0.152)	**44.15** (0.145)	58.23 (0.230)	48.06 (0.155)	48.25 (0.155)	49.98 (0.025)
0.5	49.02 (0.062)	37.73 (0.027)	35.61 (0.061)	**34.43** (0.014)	52.22 (0.207)	43.50 (0.141)	43.63 (0.142)	49.98 (0.025)
1	48.04 (0.053)	34.64 (0.015)	34.48 (0.020)	**34.43** (0.014)	45.30 (0.167)	38.73 (0.107)	39.08 (0.109)	49.98 (0.025)
5	41.19 (0.040)	**34.36** (0.017)	34.39 (0.015)	34.42 (0.015)	34.44 (0.014)	34.43 (0.014)	34.43 (0.014)	49.97 (0.025)
10	36.61 (0.020)	**34.01** (0.018)	34.18 (0.018)	34.16 (0.018)	34.43 (0.014)	34.43 (0.014)	34.43 (0.014)	49.96 (0.025)
1000	32.43 (0.009)	31.62 (0.013)	29.69 (0.017)	29.69 (0.017)	30.21 (0.014)	29.51 (0.014)	**29.48** (0.014)	47.61 (0.024)

**Figure A.1:**
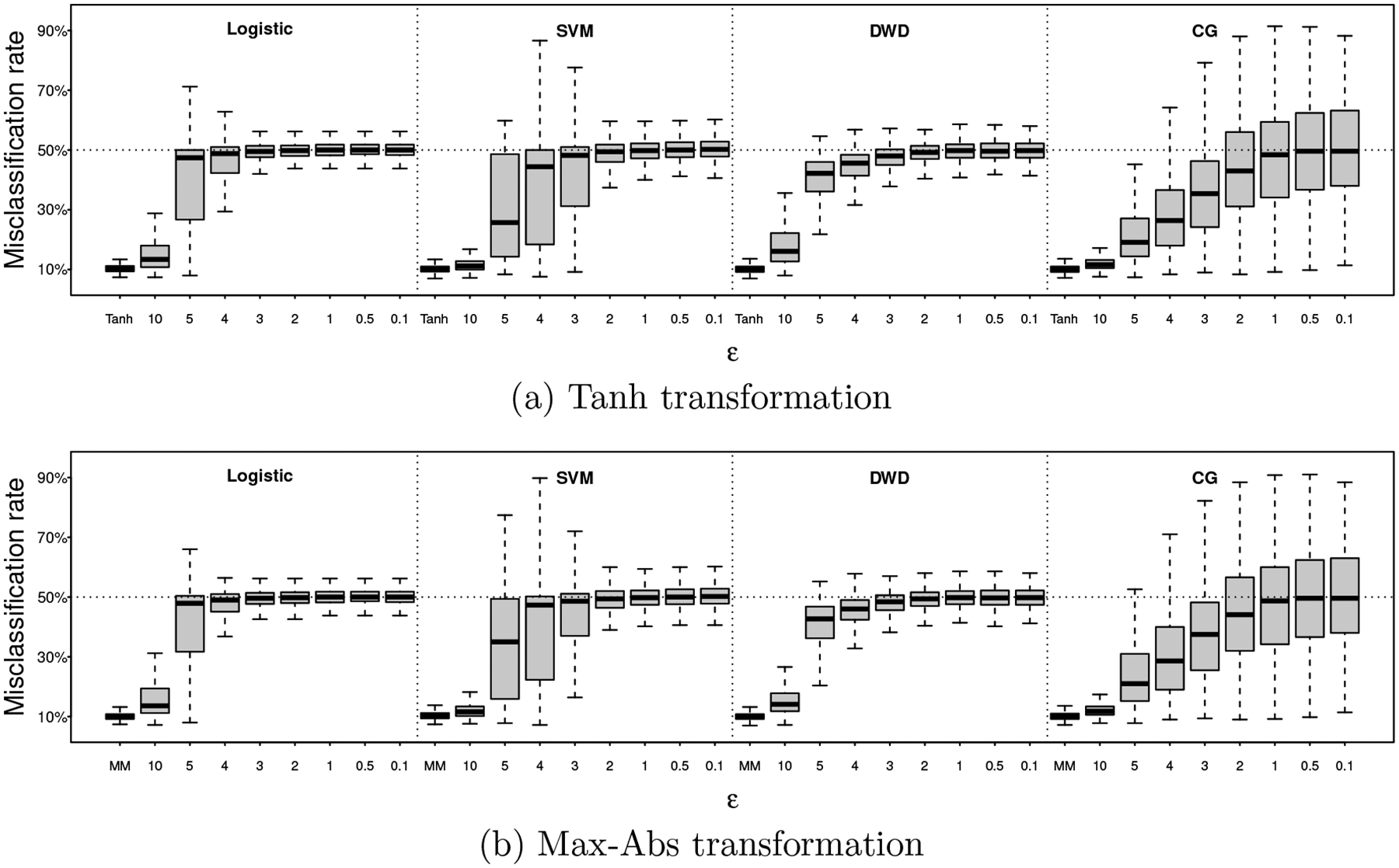
The boxplot of the misclassification rates of classifiers with Tanh and Max-Abs transformations under ε-LDP.

**Figure A.2:**
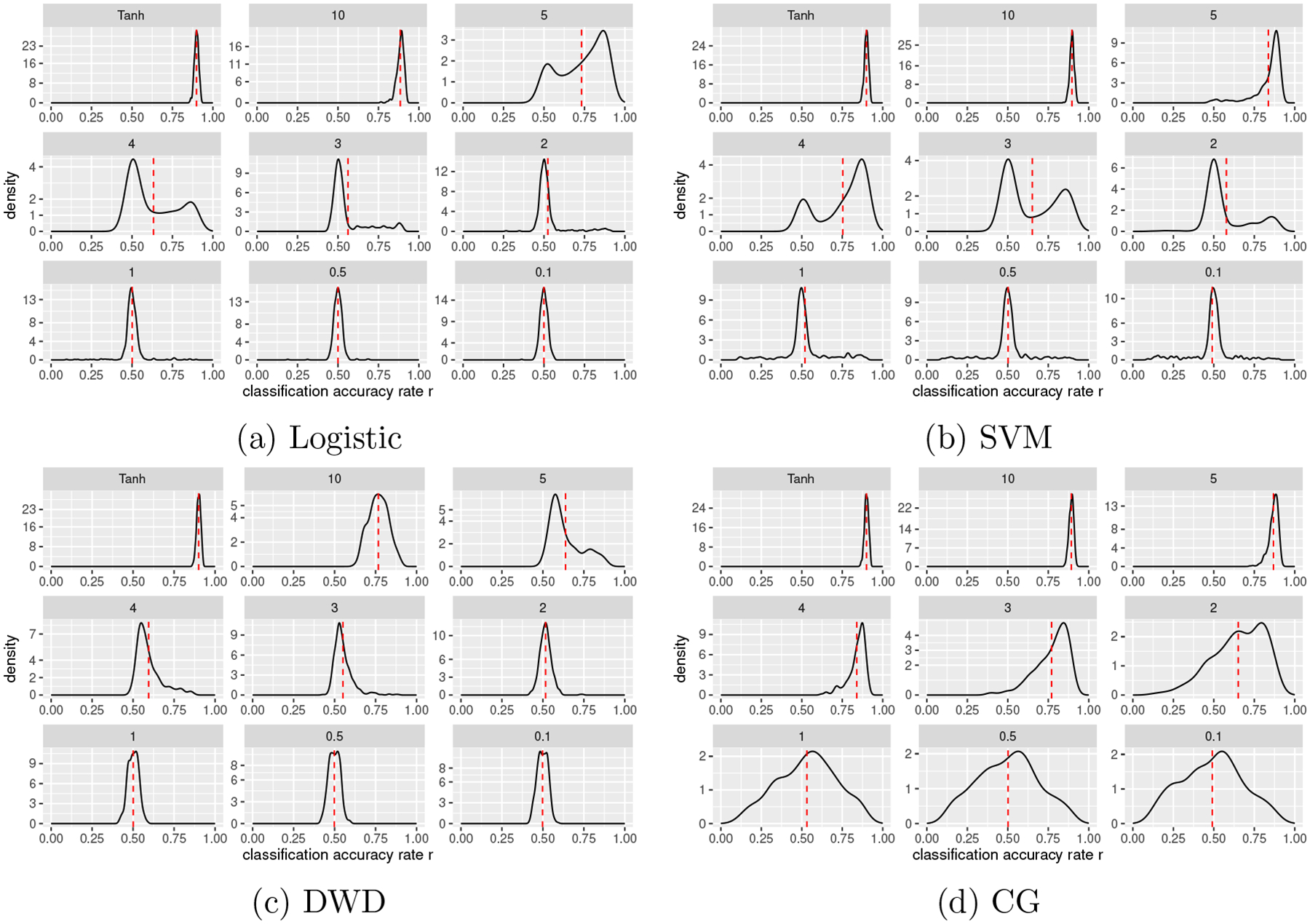
The empirical distributions of classification accuracy for classifiers trained with N=3000 clients and perturbed by Tanh transformation, where ϵ=0.1,0.5,1,…,5,10, and the red dashed line represents the mean accuracy rate.

**Figure A.3:**
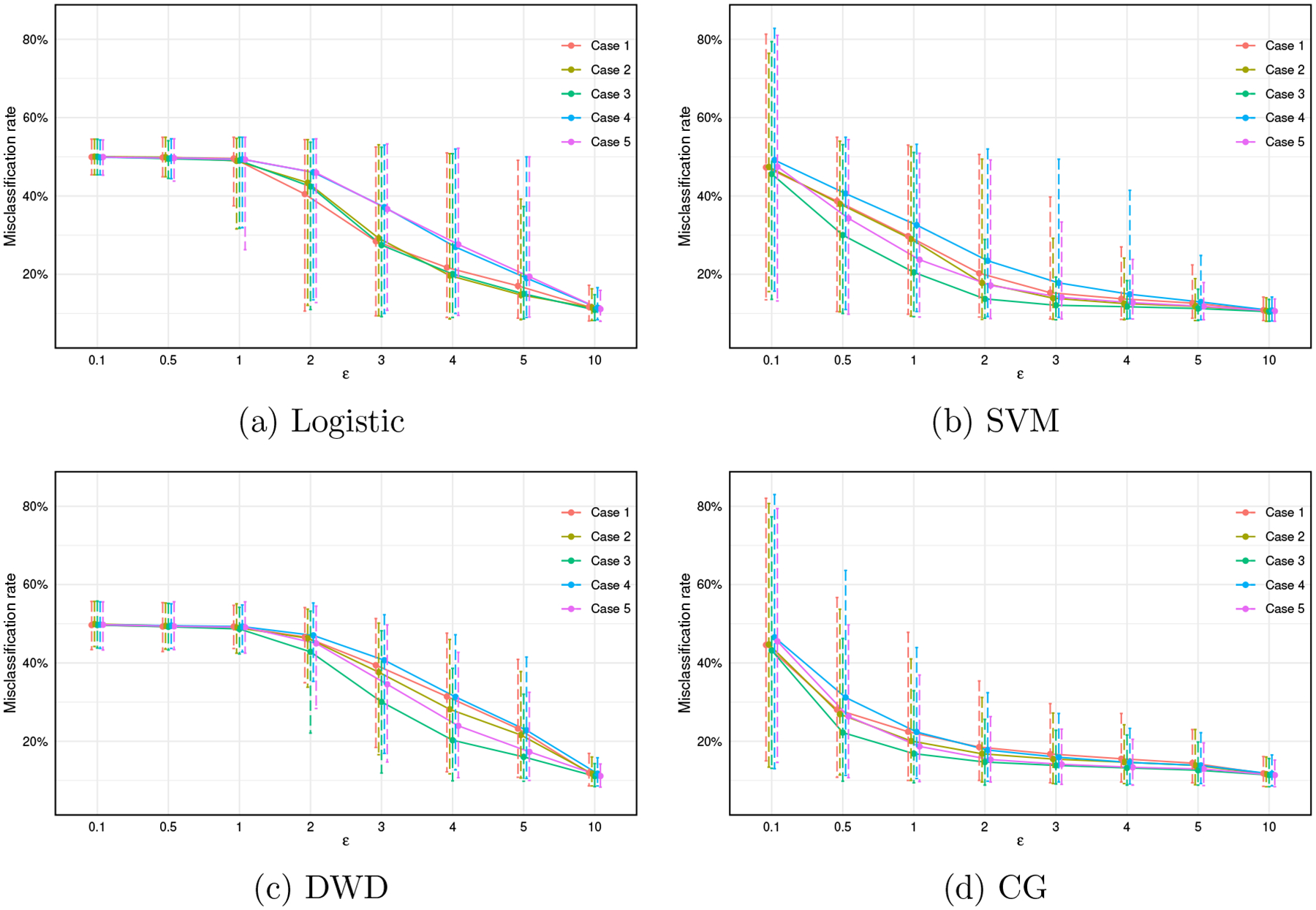
The misclassification rates classifiers with model averaging under ε-LDP.

**Figure A.4:**
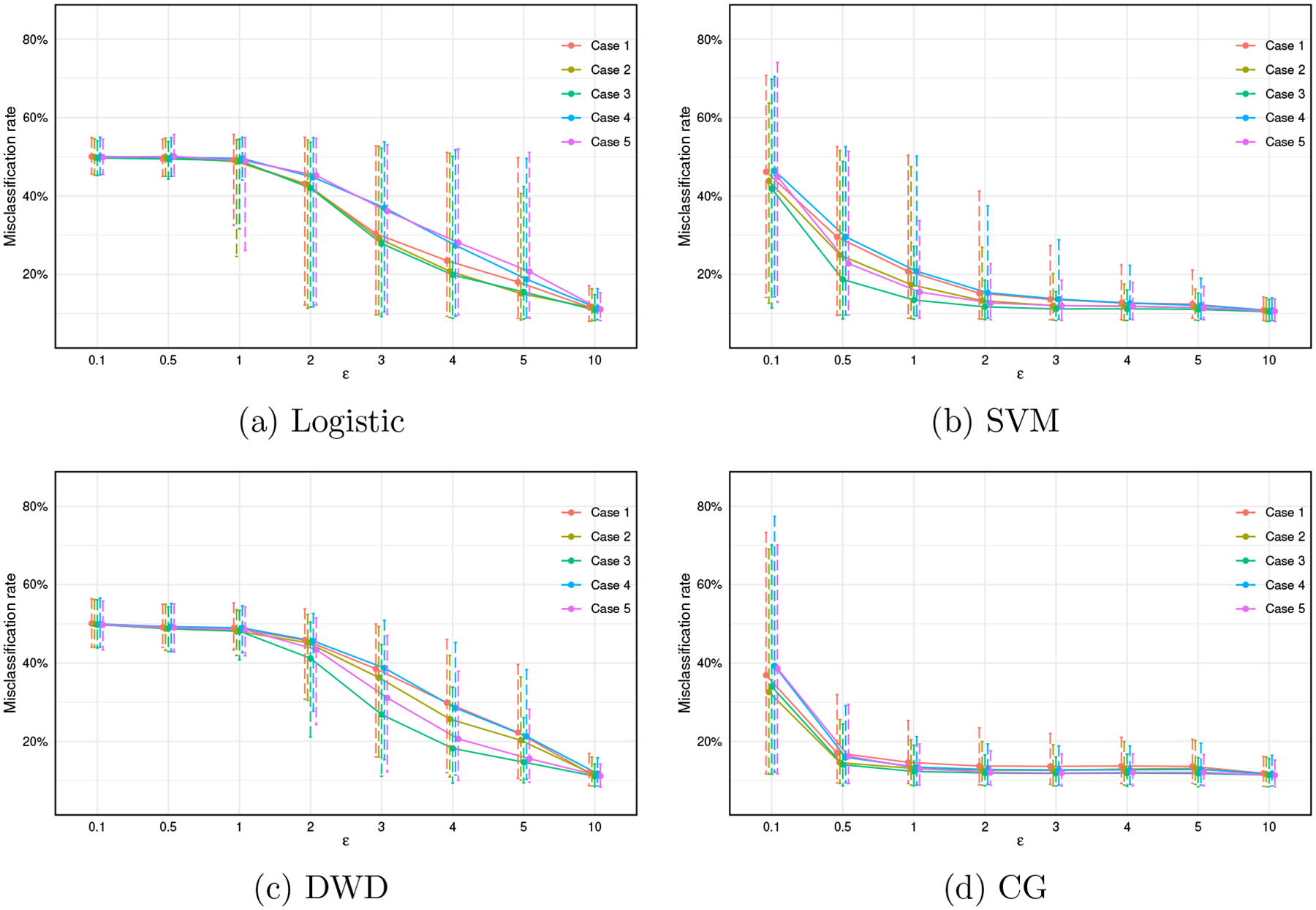
The misclassification rates of classifiers with model reversal and model averaging under ε-LDP.

**Figure A.6:**
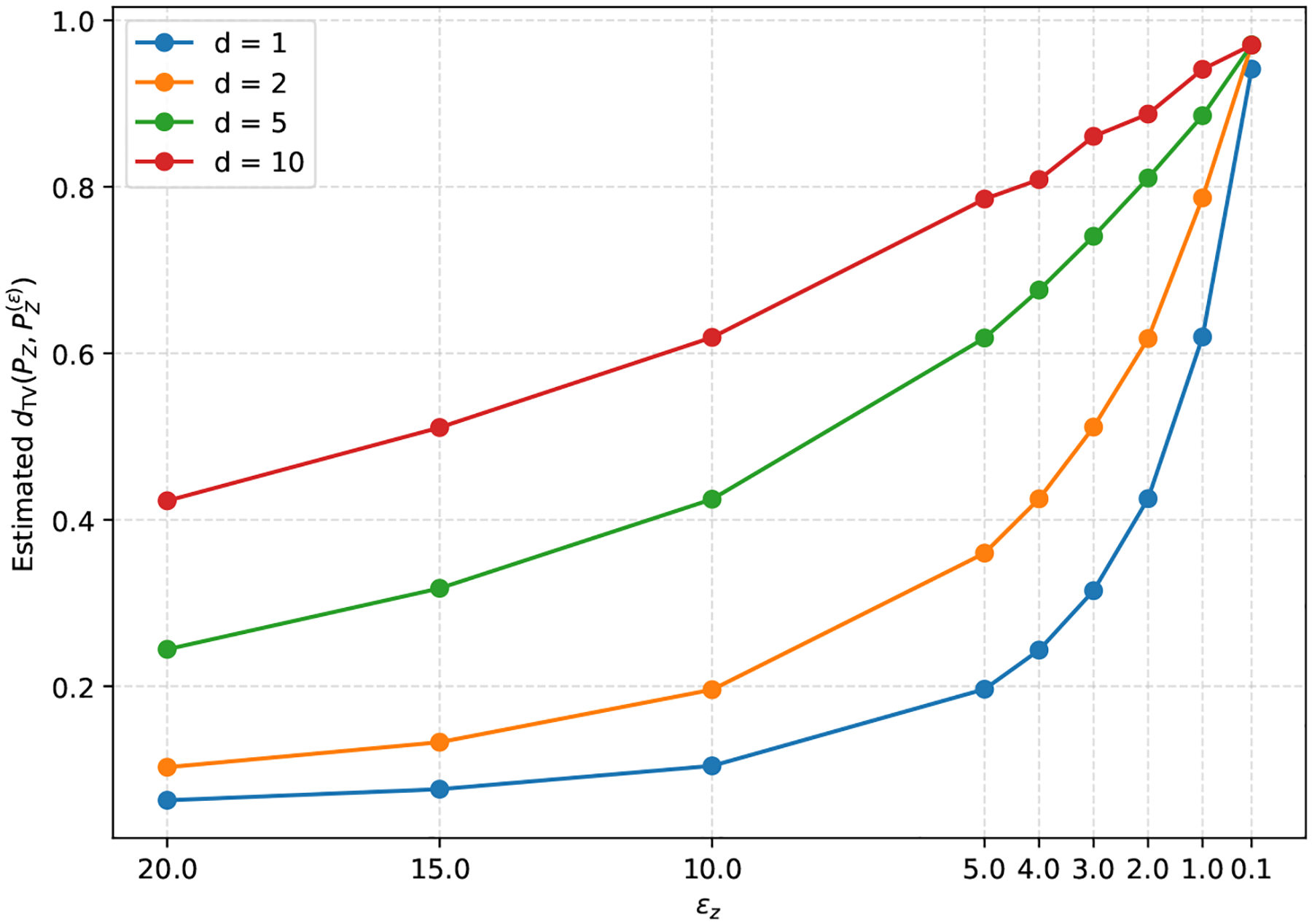
Estimated total variation distance $d_{\text{TV}}\left(P_{Z},P_{Z}^{\left(\varepsilon \right)}\right)$ under the Laplace mechanism for $Z\sim \text{Unif}\left([-1,1{]}^{d}\right)$, across dimensions d∈{1,2,5,10} and a range of privacy levels $\varepsilon_{z}$.

**Figure A.8:**
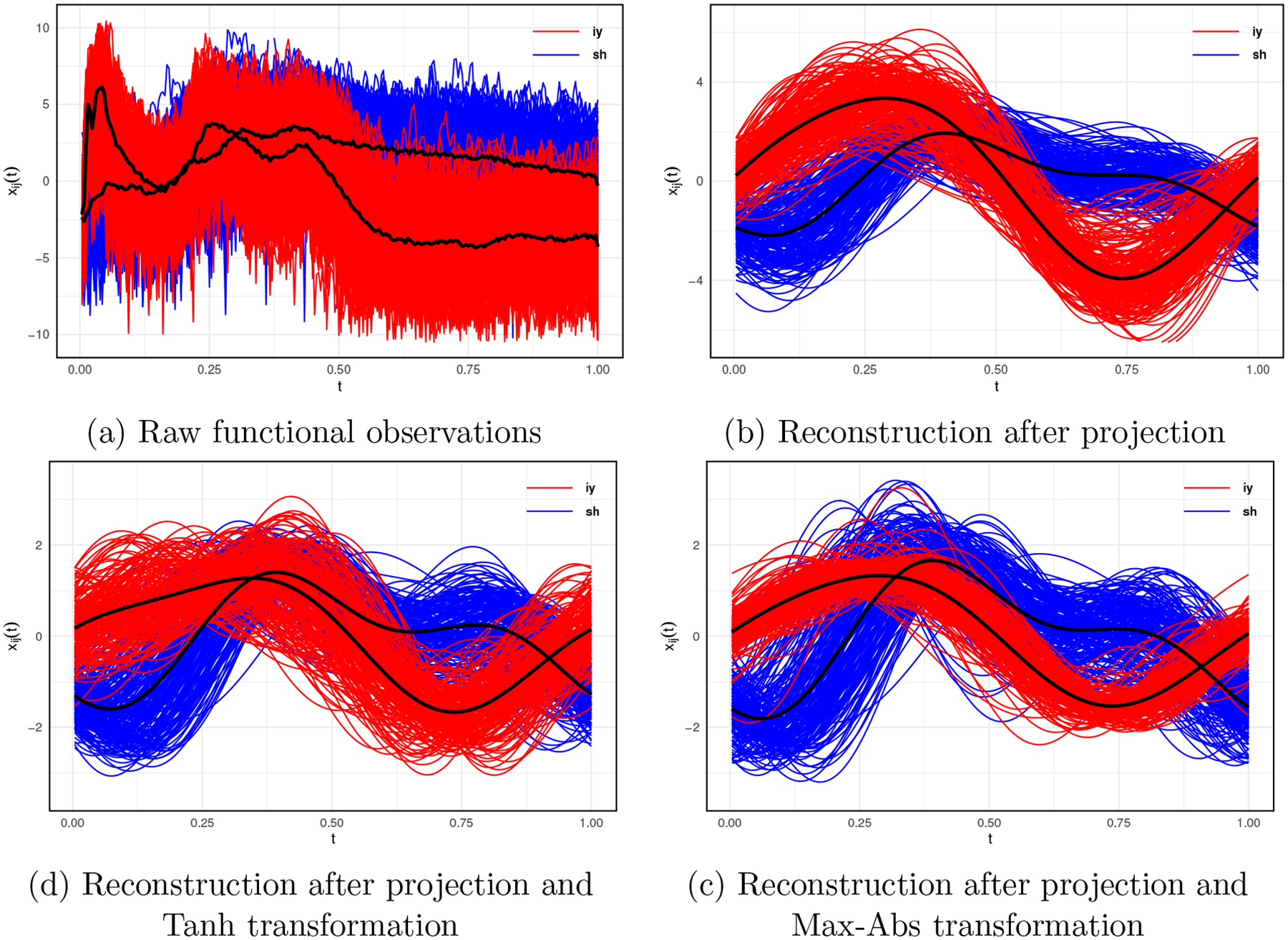
Visualization of 200 randomly selected functional observations from groups “sh” and “iy” in the phonemes data set. The two black lines in each figure represent the mean functions of the two groups of curves.

## Appendix A. Additional Results

In this section, we provide additional results from our experiments to demonstrate the impacts of encoding and perturbation, compare various sample allocation strategies, and confirm that the performance of weak classifiers improves slightly with an increase in training sample size.

### A.1 Encoding and Perturbation

In this section, we discuss the effects of dimensionality reduction, rescaling, and perturbation introduced in Section 5.1, on the misclassification rates of different types of classifiers.

To investigate the impacts of dimensionality reduction and rescaling, we generate data based on the model described in Section 6, and the sample sizes of the training and testing data sets are 50 and 500, respectively. During the dimensionality reduction, cubic B-spline with equidistant knots are employed, and we apply the methods for varying numbers of basis functions, d=4,5,6. Table A.1 showcases the misclassification rates of classifiers based on the actual data (INI), coefficients obtained after dimensionality reduction (Coefs), and coefficients rescaled by either the Tanh transformation (Tanh) or Max-Abs normalization (MAN). This is based on the results from 500 repeated experiments.

**Table A.1:** The misclassification rate of classifiers based on actual data (INI), coefficients obtained after dimensionality reduction (Coefs), and coefficients rescaled by either the Tanh transformation (Tanh) or Max-Abs normalization (MAN).

	INI	d=4			d=5			d=6		
		Coefs	Tanh	MAN	Coefs	Tanh	MAN	Coefs	Tanh	MAN
Logistic	-	17.81	12.32	11.68	20.66	13.55	12.81	23.19	15.21	13.86
SVM	-	10.85	11.20	11.03	11.29	11.67	11.27	11.69	12.07	11.48
DWD	11.01	11.02	10.64	10.61	11.01	10.60	10.58	11.01	10.61	10.59
CG	15.42	11.02	11.47	11.31	11.34	11.86	11.43	11.65	12.00	11.64

Table A.1 shows that there are slight variations in the performance of different types of classifiers. Overall, classifiers with d=4,5,6 exhibit comparable results, and the impact of dimension reduction and rescaling on classifier performance is small. Also, the performances based on Tanh and Max-Abs transformations are very similar. Furthermore, the CG classifier based on actual data and the logistic classifier based on coefficients obtained after dimensionality reduction perform relatively poorer. This may be related to the inherent characteristics of the classifiers, which is beyond the scope of this paper. Our primary focus is on the changes in classifier performance before and after consider LDP and the improvements brought about by different techniques.

To further explore the impacts of perturbation, we generate data in accordance with the model in Section 6, and both the training and testing data set sample sizes are 500. Table A.1 demonstrates that classifiers with d=4,5,6 exhibit comparable results.Therefore, we employ d=4 cubic B-spline with equidistant knots to introduce as little noise as possible. We consider eight distinct privacy budget levels, specifically, ϵ=0.1,0.5,1,…,5,10. Figure A.1 displays the misclassification rates of the four types of classifiers, based on both unperturbed rescaled coefficients (i.e., “Tanh”, “MAN”) and perturbed rescaled coefficients across various levels of ϵ.

In Figure A.1, with decreasing ε, the misclassification rates of different types of classifiers tend to 50%. At the same time, there is a variation in the performance of classifiers under the influence of noise. Notably, the misclassification rate of CG classifier remains highly volatile at smaller ε values, ranging between 10% and 90%, instead of converging around 50% as other methods do. This indicates that the performance can be enhanced through model reversal and model averaging. Additionally, it can be observed from Figure A.1 that classifiers based on both Tanh and Max-Abs transformations exhibit very similar performances. Only results based on the Tanh transformation will be presented hereafter.

In Theorem 5, we quantify how model reversal improves the excess risk bound of a classifier. In Figure A.2, we show the empirical distributions of classification accuracy for the different classifiers under our experimental settings. Figure A.2 illustrates that different classifiers exhibit varying degrees of sensitivity to noise. Among them, the DWD classifier is the most affected, followed by logistic and SVM classifiers. In contrast, the CG classifier is relatively less impacted by noise interference. As ε decreases, which corresponds to increased noise, the classification accuracy distributions for logistic, SVM, and DWD classifiers gradually converge around 0.5. However, the distribution for the CG classifier remains more dispersed, indicating greater potential for improvement through model reversal.

### A.2 Sample Size Balancing

In this section, we assess the performance of classifiers over varying values of the parameters $N,N_{0},N_{1},n_{0},n_{1},B$. We generate data based on the settings presented in Section 6. Specifically, we consider five distinct combinations of these parameter values, which are listed in Table A.2. Figures A.3 and A.4 display the misclassification rates of model-averaged classifiers using the cutoff value $r_{0}=0.6$, with and without model reversal, respectively, for the different parameter combinations.

**Table A.2:** Five different combinations of parameters $N,N_{0},N_{1},n_{0},n_{1},B$

Case	N	$N_{0}$	$N_{1}$	$n_{0}$	$n_{1}$	B
1	3000	500	2500	100	100	25
2	5500	500	5000	100	100	50
3	5500	500	5000	50	100	50
4	3000	500	2500	100	50	50
5	3000	500	2500	50	50	50

In Figure A.3, the differences arising from various parameter combinations on different types of classifiers manifest across distinct intervals of ε. Overall, Case 2 performs slightly better than Case 1, indicating that increasing the number of weak classifiers B and the sample size of the evaluation set $N_{1}$ can enhance the performance. The preference for a relatively smaller $n_{0}$ is evident as Case 3 outperforms Case 2, and Case 5 outperforms Case 4. Additionally, Case 2 is distinctly superior to Case 4, and Case 3 is markedly better than Case 5, indicating a preference for a larger $n_{1}$. Importantly, by comparing Case 1 and Case 4, we discern that, given $N_{0}$ and $N_{1}$, the allocation should favor a relatively smaller B and a larger $n_{1}$. This result is consistent with expectations, as a larger $n_{1}$ aids in more accurately estimating the performance of individual weak classifiers, thereby facilitating a more accurate model averaging.

Compared to Figure A.3, Figure A.4 incorporates model reversal prior to model averaging. It can be observed that the results of the various parameter combinations in Figure A.4 are consistent with those in Figure A.3. Notably, the introduction of model reversal has significantly enhanced the performance of both SVM and CG classifiers, particularly under more stringent privacy protection levels, characterized by smaller intervals of ε.

### A.3 Sample Size per Weak Classifier

To demonstrate how the number of clients used to train weak classifiers influences the efficacy of MRMA in improving classifier performance, we consider four distinct settings, which are listed in Table A.3. Figure A.5 shows how the misclassification rates of the classifier CG vary with different parameter settings.

We can see from the results of cases 5, 6, and 7 in Figure A.5 that using more clients to train weak classifiers improves their performance when ε is large, implying low noise levels. However, when ε is small, which is our primary concern, increasing the sample size has little effect on the weak classifiers. In contrast, the classifiers based on MRMA consistently achieve significant improvements under different cases. Moreover, when we compare cases 7 and 8 in Figure A.5, where the training and evaluation data sets have different proportions, we find that allocating more data for evaluation, i.e., for MRMA, leads to better results than using it to enhance weak classifiers.

**Table A.3:** Four different combinations of parameters $N,N_{0},N_{1},n_{0},n_{1},B$

Case	N	$N_{0}$	$N_{1}$	$n_{0}$	$n_{1}$	B
5	3000	500	2500	50	50	50
6	5000	2500	2500	250	50	50
7	7500	5000	2500	500	50	50
8	7500	2500	5000	250	100	50

### A.4 Numerical Illustration of the Total Variation Distance

The excess risk bounds in Theorems 4–6 involve the term $d_{\text{TV}}\left(P_{Z},P_{Z}^{\left(\varepsilon \right)}\right)$, which measures the discrepancy between the marginal distributions of Z and its privatized version $Z^{\left(\varepsilon_{z}\right)}$. Closed-form expressions for this total variation distance are generally unavailable, even under simple additive-noise mechanisms. To provide intuition regarding the behavior of this term, we present a simple numerical illustration.

We consider $Z\sim \text{Unif}\left([-1,1{]}^{d}\right)$ and apply the Laplace mechanism (Dwork et al., 2006), which adds i.i.d. noise $\delta_{k}\sim \text{Laplace}\left(0,\lambda_{z}\right)$ to each coordinate, where $\lambda_{z}=d(b-a)/\varepsilon_{z}$ with [a,b]=[-1,1]. The distribution of $Z^{\left(\varepsilon_{z}\right)}=Z+\delta$ typically has no simple closed form, so we approximate $d_{\text{TV}}\left(P_{Z},P_{Z}^{\left(\varepsilon \right)}\right)$ via Monte Carlo: the empirical distributions are estimated on a common grid, and the total variation distance is computed as

$$d_{\text{TV}}\left(P,Q\right)\approx \frac{1}{2}\sum_{k}\left|{\overset{ˆ}{P}}_{k}-{\overset{ˆ}{Q}}_{k}\right|.$$

Figure A.6 reports the estimated values for several dimensions d∈{1,2,5,10} and a range of privacy levels $\varepsilon_{z}$. The results reveal two intuitive patterns. First, as $\varepsilon_{z}\to 0$ (i.e., stronger privacy and heavier noise), the discrepancy between $P_{Z}$ and $P_{Z}^{\left(\varepsilon \right)}$ increases, and the total variation distance approaches 1. This corresponds to the case where the privatized distribution becomes almost disjoint from the original distribution. Second, for fixed $\varepsilon_{z}$, the distance increases with the dimension d, because the per-coordinate privacy budget effectively decreases as d grows. This is reflected in the scale parameter $\lambda_{z}=d(b-a)/\varepsilon_{z}$, which increases with d when the total privacy level $\varepsilon_{z}$ is fixed, and thus induces larger Laplace noise in each coordinate.

### A.5 Visualization of Functional Predictors

Figures A.7–A. 8 provide visualizations of the functional predictors used in the physical activity and speech datasets, respectively, under different stages of processing: raw functional data, basis projection, and two types of transformations. In Figure A.7, we display the group mean MIMS curves for participants with healthy (HDL ≥ 50) and unhealthy (HDL < 50) cholesterol levels, along with 10 randomly selected individual curves shown in gray. We limit the number of individual curves to reduce visual clutter, as the raw MIMS functions exhibit substantial variability across participants. Figure A.8 presents 200 randomly selected individual log-periodogram curves from the speech dataset along with the group means. Despite some smoothing and loss of fine-scale detail, these visualizations demonstrate that both temporal fluctuations and population-level differences are preserved throughout the functional representation pipeline.

## Appendix B. Theoretical Analysis

**Proof** [of Proposition 2] We begin by noting that $g^{\left(\varepsilon \right)}\left(z_{0}\right)=\eta \left(z_{0}\right)\eta^{\left(\varepsilon \right)}\left(z_{0}\right)+\left(1-\eta \left(z_{0}\right)\right)(1-\eta^{\left(\varepsilon \right)}\left(z_{0}\right))$, which yields the first expression in Equation (3).

To compute $\eta^{\left(\varepsilon \right)}\left(z_{0}\right)-\frac{1}{2}$, we have that

$$\eta^{\left(\varepsilon \right)}\left(z_{0}\right)=\frac{P^{\left(\varepsilon \right)}\left(Z^{\left(\varepsilon_{z}\right)}=z_{0},Y^{\left(\varepsilon_{y}\right)}=1\right)}{P_{Z}^{\left(\varepsilon \right)}\left(Z^{\left(\varepsilon_{z}\right)}=z_{0}\right)},$$

where

$$P_{Z}^{\left(\varepsilon \right)}\left(Z^{\left(\varepsilon_{z}\right)}=z_{0}\right)=E_{P_{Z}}\left(D^{\left(\varepsilon_{z}\right)}\left(\delta_{z}^{\left(\varepsilon_{z}\right)}=z_{0}-z\right)\right),\;P^{\left(\varepsilon \right)}\left(Z^{\left(\varepsilon_{z}\right)}=z_{0},Y^{\left(\varepsilon_{y}\right)}=1\right)=E_{P_{Z}}\left(D^{\left(\varepsilon_{z}\right)}\left(\delta_{z}^{\left(\varepsilon_{z}\right)}=z_{0}-z\right)\left(q^{\left(\varepsilon_{y}\right)}\eta (z)+\left(1-q^{\left(\varepsilon_{y}\right)}\right)(1-\eta (z))\right)\right).$$

After some algebraic manipulation, we arrive at the expression given in Equation (4). ■

**Proof** [of Theorem 3] Let u=(x,y) represent the possible observation of a client, and $w=r^{\prime }$ represent the possible report of a client. Let $f_{w|u}(w|u)$ be the conditional density of w given u. Then for any w∈{0,1}, we have

$$\frac{f_{w|u}\left(w|u_{1}\right)}{f_{w|u}\left(w|u_{2}\right)}=\frac{P\left(r^{\prime }|r_{1}\right)}{P\left(r^{\prime }|r_{2}\right)}\leq e^{\varepsilon_{v}}.$$

Thus ℳ satisfies $\varepsilon_{v}$-local differential privacy. Note that $r^{\left(b\right)}$ is the classification accuracy of the classifier $f^{\left(b\right)}$, then

$$E\left({\overset{ˆ}{r}}^{\left(b\right)}\right)=qr^{\left(b\right)}+(1-q)\left(1-r^{\left(b\right)}\right),$$

where $q=e^{\varepsilon_{v}}/\left(1+e^{\varepsilon_{v}}\right)$. Thus we have $E\left({\tilde{r}}^{\left(b\right)}\right)=r^{\left(b\right)}$, and $\text{Var}\left({\tilde{r}}^{\left(b\right)}\right)=\text{Var}\left({\overset{ˆ}{r}}^{\left(b\right)}\right)/(2q-1{)}^{2}\leq {\left(\left(e^{\varepsilon_{v}}+1\right)/\left(e^{\varepsilon_{v}}-1\right)\right)}^{2}/\left(4n_{1}^{\left(b\right)}\right)$. This concludes the proof. ■

**Proof** [of Theorem 4] By definition, we have

$$L_{P}\left(f^{\left(b\right)},h^{\text{*}}\right)=E_{P}\left\{\ell \left(f^{\left(b\right)}\right)-\ell \left(h^{\text{*}}\right)\right\}\;=\left\{E_{P}\ell \left(f^{\left(b\right)}\right)-E_{P^{\left(\varepsilon \right)}}\ell \left(f^{\left(b\right)}\right)\right\}+E_{P^{\left(\varepsilon \right)}}\left\{\ell \left(f^{\left(b\right)}\right)-\ell \left(h^{\text{*}\left(\varepsilon \right)}\right)\right\}+\left\{E_{P^{\left(\varepsilon \right)}}\ell \left(h^{\text{*}\left(\varepsilon \right)}\right)-E_{P}\ell \left(h^{\text{*}}\right)\right\}\;≜I_{1}+I_{2}+I_{3}.$$

To analyze $I_{1}=E_{P}\ell \left(f^{\left(b\right)}\right)-E_{P^{\left(\varepsilon \right)}}\ell \left(f^{\left(b\right)}\right)$, let p(z) and $p^{\left(\varepsilon \right)}(z)$ denote the density functions of Z and $Z^{\left(\varepsilon \right)}$, respectively. Define

$$p_{0}(z)=\text{min}\left\{p(z),p^{\left(\varepsilon \right)}(z)\right\},\;p_{1}(z)=p(z)-p_{0}(z),\;p_{1}^{\left(\varepsilon \right)}(z)=p^{\left(\varepsilon \right)}(z)-p_{0}(z).$$

Then,

$$I_{1}=E_{P_{Z}}\left\{\eta \left(z\right)\ell \left(f^{\left(b\right)}|y=1\right)+\left(1-\eta \left(z\right)\right)\ell \left(f^{\left(b\right)}|y=-1\right)\right\}\;-E_{P_{Z}^{\left(\varepsilon z\right)}}\left\{\eta^{\left(\varepsilon \right)}\left(z\right)\ell \left(f^{\left(b\right)}|y=1\right)+\left(1-\eta^{\left(\varepsilon \right)}\left(z\right)\right)\ell \left(f^{\left(b\right)}|y=-1\right)\right\}\;=\int p_{0}\left(z\right)\left\{\eta \left(z\right)-\eta^{\left(\varepsilon \right)}\left(z\right)\right\}\left\{\ell \left(f^{\left(b\right)}|y=1\right)-\ell \left(f^{\left(b\right)}|y=-1\right)\right\}dz\;+\int p_{1}\left(z\right)\ell \left(f^{\left(b\right)}\right)dz-\int p_{1}^{\left(\varepsilon \right)}\left(z\right)\ell \left(f^{\left(b\right)}\right)dz\;\leq E_{P_{Z}}\left\{\left|\eta \left(z\right)-\eta^{\left(\varepsilon \right)}\left(z\right)\right|\right\}+\int p_{1}\left(z\right)dz.$$

Next, consider $I_{3}=E_{P^{\left(\varepsilon \right)}}\ell \left(h^{*(\varepsilon )}\right)-E_{P}\ell \left(h^{*}\right)$. Note that

$$E_{P}\ell \left(h^{\text{*}}\right)=E_{P_{Z}}\left\{I\left\{\eta \left(z\right)\geq 1/2\right\}P_{Y|Z}\left(y=-1\right)+I\{\eta \left(z\right)<1/2\}P_{Y|Z}\left(y=1\right)\right\}\;=E_{P_{Z}}\{I\left\{\eta \left(z\right)\geq 1/2\right\}\left(1-\eta \left(z\right)\right)+I\{\eta \left(z\right)<1/2\}\eta \left(z\right)\}\;=E_{P_{Z}}\left\{\frac{1}{2}-\left|\eta \left(z\right)-\frac{1}{2}\right|\right\},$$

and similarly,

$$E_{P^{\left(\varepsilon \right)}}\ell \left(h^{*(\varepsilon )}\right)=E_{P_{Z}^{\left(\varepsilon \right)}}\left\{\frac{1}{2}-\left|\eta^{\left(\varepsilon \right)}(z)-\frac{1}{2}\right|\right\}.$$

Thus,

$$I_{3}=E_{P_{Z}}\left\{\left|\eta \left(z\right)-\frac{1}{2}\right|\right\}-E_{P_{Z}^{\left(\varepsilon \right)}}\left\{\left|\eta^{\left(\varepsilon \right)}\left(z\right)-\frac{1}{2}\right|\right\}\;=\int p_{0}\left(z\right)\left\{\left|\eta \left(z\right)-\frac{1}{2}\right|-\left|\eta^{\left(\varepsilon \right)}\left(z\right)-\frac{1}{2}\right|\right\}dz\;+\int p_{1}\left(z\right)\left|\eta \left(z\right)-\frac{1}{2}\right|dz-\int p_{1}^{\left(\varepsilon \right)}\left(z\right)\left|\eta^{\left(\varepsilon \right)}\left(z\right)-\frac{1}{2}\right|dz\;\leq E_{P_{Z}}\left\{\left|\eta \left(z\right)-\frac{1}{2}\right|-\left|\eta^{\left(\varepsilon \right)}\left(z\right)-\frac{1}{2}\right|\right\}+\frac{1}{2}\int p_{1}\left(z\right)dz.$$

By the definition of total variation distance, we have

$$d_{\text{TV}}\left(P_{Z},P_{Z}^{\left(\varepsilon \right)}\right)=2\int p_{1}\left(z\right)dz.$$

Combining the bounds for $I_{1}$ and $I_{3}$, we obtain the result stated in the theorem. ■

**Proof** [of Theorem 5] Consider the noised dataset ${\left\{\left(z_{i}^{\left(\varepsilon_{z}\right)},y_{i}^{\left(\varepsilon_{y}\right)}\right)\right\}}_{i=1}^{n_{0}}$ and the classifier $f^{\left(b\right)}$ trained on it. We define a pseudo-noised dataset ${\left\{\left(z_{i}^{\left(\varepsilon_{z}\right)},{\tilde{y}}_{i}^{\left(\varepsilon_{y}\right)}\right)\right\}}_{i=1}^{n_{0}}$ where ${\tilde{y}}_{i}^{\left(\varepsilon_{y}\right)}=-y_{i}^{\left(\varepsilon_{y}\right)}$, and correspondingly define ${\tilde{f}}^{\left(b\right)}=-f^{\left(b\right)}$. Then ${\tilde{f}}^{\left(b\right)}$ can be interpreted as the classifier trained on this pseudo-noised dataset.

Let ${\tilde{P}}^{\left(\varepsilon \right)}$ denote the joint distribution of $\left(Z^{\left(\varepsilon_{z}\right)},{\tilde{Y}}^{\left(\varepsilon_{y}\right)}\right)$. Then the marginal distribution of $Z^{\left(\varepsilon_{z}\right)}$ under ${\tilde{P}}^{\left(\varepsilon \right)}$ remains the same as that under $P^{\left(\varepsilon \right)}$, i.e., ${\tilde{P}}_{Z}^{\left(\varepsilon \right)}=P_{Z}^{\left(\varepsilon \right)}$. The conditional distribution satisfies ${\tilde{P}}_{Y|Z}^{\left(\varepsilon \right)}=1-P_{Y|Z}^{\left(\varepsilon \right)}$, and the corresponding Bayes classifier becomes ${\tilde{h}}^{*(\varepsilon )}(z)=\text{sign}\left\{{\tilde{\eta }}^{\left(\varepsilon \right)}(z)\geq 1/2\right\}=-h^{*(\varepsilon )}(z)$, where ${\tilde{\eta }}^{\left(\varepsilon \right)}(z)=1-\eta^{\left(\varepsilon \right)}(z)$.

Applying Theorem 4, we obtain

$$L_{P}\left(-f^{\left(b\right)},h^{*}\right)=L_{P}\left({\tilde{f}}^{\left(b\right)},h^{*}\right)\leq L_{{\tilde{P}}^{\left(\varepsilon \right)}}\left({\tilde{f}}^{\left(b\right)},{\tilde{h}}^{*(\varepsilon )}\right)+\frac{3}{4}d_{\text{TV}}\left(P_{Z},{\tilde{P}}_{Z}^{\left(\varepsilon \right)}\right)+E_{P_{Z}}\left(\left|\eta (z)-{\tilde{\eta }}^{\left(\varepsilon \right)}(z)\right|\right)\;+E_{P_{Z}}\left(\left|\eta (z)-\frac{1}{2}\right|-\left|{\tilde{\eta }}^{\left(\varepsilon \right)}(z)-\frac{1}{2}\right|\right)\;=L_{P^{\left(\varepsilon \right)}}\left(f^{\left(b\right)},h^{*(\varepsilon )}\right)+\frac{3}{4}d_{\text{TV}}\left(P_{Z},P_{Z}^{\left(\varepsilon \right)}\right)+E_{P_{Z}}\left(\left|\eta (z)-{\tilde{\eta }}^{\left(\varepsilon \right)}(z)\right|\right)\;+E_{P_{Z}}\left(\left|\eta (z)-\frac{1}{2}\right|-\left|\eta^{\left(\varepsilon \right)}(z)-\frac{1}{2}\right|\right).$$

Furthermore, we note that

$$\text{min}\left\{E_{P_{Z}}\left(\left|\eta (z)-{\tilde{\eta }}^{\left(\varepsilon \right)}(z)\right|\right),E_{P_{Z}}\left(\left|\eta (z)-\eta^{\left(\varepsilon \right)}(z)\right|\right)\right\}=E_{P_{Z}}\left(\left|\eta (z)-\frac{1}{2}\right|-\left|\eta^{\left(\varepsilon \right)}(z)-\frac{1}{2}\right|\right).$$

This implies

$$\text{min}\left\{L_{P}\left(f^{\left(b\right)},h^{*}\right),L_{P}\left(-f^{\left(b\right)},h^{*}\right)\right\}\leq L_{P^{\left(\varepsilon \right)}}\left(f^{\left(b\right)},h^{*(\varepsilon )}\right)+\frac{3}{4}d_{\text{TV}}\left(P_{Z},P_{Z}^{\left(\varepsilon \right)}\right)+2E_{P_{Z}}\left(\left|\eta (z)-\frac{1}{2}\right|-\left|\eta^{\left(\varepsilon \right)}(z)-\frac{1}{2}\right|\right).$$

Let $r^{\left(b\right)}$ denote the true classification accuracy of the Bayes classifier $h^{*(\varepsilon )}$ under the distribution P, and let ${\tilde{r}}^{\left(b\right)}$ denote the estimated classification accuracy of $f^{\left(b\right)}$ based on $n_{1}$ evaluation samples. Define the selected classifier $f^{*(b)}\in \left\{f^{\left(b\right)},-f^{\left(b\right)}\right\}$ as the one with higher estimated accuracy: specifically, $f^{*(b)}=f^{\left(b\right)}$ if ${\overset{ˆ}{c}}_{n_{1}}\geq 1/2$, and $f^{*(b)}=-f^{\left(b\right)}$ otherwise. Then, the probability that model reversal correctly selects the better classifier satisfies

$$P\left[L_{P}\left(f^{*(b)},h^{*}\right)=\text{min}\left\{L_{P}\left(f^{\left(b\right)},h^{*}\right),L_{P}\left(-f^{\left(b\right)},h^{*}\right)\right\}\right]=P\left\{\left({\tilde{r}}^{\left(b\right)}-\frac{1}{2}\right)\left(r^{\left(b\right)}-\frac{1}{2}\right)\geq 0\right\}\;\approx \Phi \left\{\sqrt{n_{1}}\frac{\left|r^{\left(b\right)}-1/2\right|}{\sqrt{r^{\left(b\right)}\left(1-r^{\left(b\right)}\right)}}\right\},$$

where Φ is the cumulative distribution function of the standard normal distribution. ■

**Proof** [of Theorem 6] Let $f^{†}(z)=\sum_{b=1}^{B}w_{b}f^{*(b)}(z)$ be the model-averaged classifier, where each $f^{*(b)}\in \left\{f^{\left(b\right)},-f^{\left(b\right)}\right\}$ is selected via model reversal to maximize estimated accuracy, and the weights ${\left\{w_{b}\right\}}_{b=1}^{B}$ sum to 1. Let ${\tilde{r}}_{z}^{*(b)}=P\left\{\text{sign}\left[f^{*(b)}(z)\right]=y\right\}$ denote the classification accuracy of the b-th classifier at point z. Let $F_{z}^{\left(\varepsilon \right)}$ be the distribution of ${\tilde{r}}_{z}^{*(b)}$ under random training and evaluation sets, with support $\left[r_{z,0},r_{z,1}\right]$. Define the aggregated mapping

$$\eta^{†\left(\varepsilon \right)}\left(z\right)≔\sum_{b=1}^{B}w_{b}\eta^{*\left(b\right)}\left(z\right),$$

where $\eta^{*(b)}(z)≔P\left\{Y=1|Z=z,f^{*(b)}\right\}$ is the latent regression function implied by classifier $f^{*(b)}$. Under standard assumptions, the classification accuracy of $f^{*(b)}(z)$ is

$${\tilde{r}}_{z}^{*(b)}=\frac{1}{2}+\left|\eta^{*(b)}(z)-\frac{1}{2}\right|.$$

As B→∞, the empirical average of classifiers concentrates around those with higher accuracy, due to the weight truncation scheme that sets $w_{b}=0$ if ${\tilde{r}}_{z}^{*(b)}<r_{0}$. Since the condition $B_{0}/B\to 0$ ensures that only a vanishing fraction of classifiers fall below the cutoff $r_{0}$, the weighted average function $\eta^{†(\varepsilon )}(z)$ satisfies

$$\left|\eta^{†(\varepsilon )}(z)-\frac{1}{2}\right|\overset{P\;}{\to }r_{z,1}-\frac{1}{2}.$$

Now, recall from Theorem 5 that for each classifier $f^{*(b)}$, the excess risk is bounded by $L_{P}\left(f^{*(b)},h^{*}\right)\leq L_{P^{\left(\varepsilon \right)}}\left(f^{*(b)},h^{*(\varepsilon )}\right)+\frac{3}{4}d_{\text{TV}}\left(P_{Z},P_{Z}^{\left(\varepsilon \right)}\right)+2E_{P_{Z}}\left(\left|\eta (z)-\frac{1}{2}\right|-\left|\eta^{*(b)}(z)-\frac{1}{2}\right|\right)$.

Taking a convex combination over b, and using the fact that the minimum excess risk over $\left\{f^{*(b)}\right\}$ is preserved in expectation under model averaging, we obtain

$$L_{P}\left(f^{†},h^{*}\right)\leq L_{P^{\left(\varepsilon \right)}}\left(f^{†},h^{*(\varepsilon )}\right)+\frac{3}{4}d_{\text{TV}}\left(P_{Z},P_{Z}^{\left(\varepsilon \right)}\right)+2E_{P_{Z}}\left(\left|\eta (z)-\frac{1}{2}\right|-\left|\eta^{†,(\varepsilon )}(z)-\frac{1}{2}\right|\right).$$

As discussed, $\left|\eta^{†,(\varepsilon )}(z)-\frac{1}{2}\right|\to r_{z,1}-\frac{1}{2}$ in probability, which establishes the theorem. ■

**Proof** [of Theorem 7] Let u=(x(t),y) represent the possible observation of a client, and $v=\left(z^{\left(\varepsilon_{z}\right)},y^{\left(\varepsilon_{y}\right)}\right)$ represent the possible report of a client. Let $f_{v|u}(v|u)$ be the conditional density of v given u. Then for any possible output v of ${ℳ}_{f}(u)$, by the sequential composition theorem (McSherry and Talwar, 2007), we have

$$\frac{f_{v|u}\left(v|u_{1}\right)}{f_{v|u}\left(v|u_{2}\right)}=\frac{P\left(y^{\left(\varepsilon_{y}\right)}|y_{1}\right)}{P\left(y^{\left(\varepsilon_{y}\right)}|y_{2}\right)}\prod_{k=1}^{d}\frac{f\left(z_{k}^{\left(\varepsilon_{z}\right)}-z_{1,k}^{*}\right)}{f\left(z_{k}^{\left(\varepsilon_{z}\right)}-z_{2,k}^{*}\right)}\;\leq \text{max}\left(1,\frac{q}{1-q},\frac{1-q}{q}\right)\prod_{k=1}^{d}\text{exp}\left(\frac{\varepsilon_{z}}{d\Delta }\left(\left|z_{k}^{\left(\varepsilon_{z}\right)}-z_{1,k}^{*}\right|-\left|z_{k}^{\left(\varepsilon_{z}\right)}-z_{2,k}^{*}\right|\right)\right)\;\leq e^{\varepsilon_{y}}\prod_{k=1}^{d}\text{exp}\left(\frac{\varepsilon_{z}}{d}\right)=e^{\varepsilon_{z}+\varepsilon_{y}}=e^{\varepsilon },$$

where $q=e^{\varepsilon_{y}}/\left(1+e^{\varepsilon_{y}}\right)$. Thus ${ℳ}_{f}$ satisfies ε-local differential privacy. ■
